# Single-cell RNA-seq reveals cellular heterogeneity from deep fascia in patients with acute compartment syndrome

**DOI:** 10.3389/fimmu.2022.1062479

**Published:** 2023-01-18

**Authors:** Tao Wang, Yubin Long, Lijie Ma, Qi Dong, Yiran Li, Junfei Guo, Lin Jin, Luqin Di, Yingze Zhang, Ling Wang, Zhiyong Hou

**Affiliations:** ^1^ Department of Orthopaedic Surgery, Third Hospital of Hebei Medical University, Shijiazhuang, Hebei, China; ^2^ Orthopaedic Research Institute of Hebei Province, Shijiazhuang, Hebei, China; ^3^ National Health Commission (NHC) Key Laboratory of Intelligent Orthopaedic Equipment, The Third Hospital of Hebei Medical University, Shijiazhuang, Hebei, China; ^4^ Department of Orthopedic Oncology, The Third Hospital of Hebei Medical University, Shijiazhuang, Hebei, China

**Keywords:** acute compartment syndrome, single cell RNA seq, immune cell, fibroblast, heat shock protein

## Abstract

**Introduction:**

High stress in the compartment surrounded by the deep fascia can cause acute compartment syndrome (ACS) that may result in necrosis of the limbs. The study aims to investigate the cellular heterogeneity of the deep fascia in ACS patients by single-cell RNA sequencing (scRNA-seq).

**Methods:**

We collected deep fascia samples from patients with ACS (high-stress group, HG, n=3) and patients receiving thigh amputation due to osteosarcoma (normal-stress group, NG, n=3). We utilized ultrasound and scanning electron microscopy to observe the morphologic change of the deep fascia, used multiplex staining and multispectral imaging to explore immune cell infiltration, and applied scRNA-seq to investigate the cellular heterogeneity of the deep fascia and to identify differentially expressed genes.

**Results:**

Notably, we identified GZMK^+^interferon-act CD4 central memory T cells as a specific high-stress compartment subcluster expressing interferon-related genes. Additionally, the changes in the proportions of inflammation-related subclusters, such as the increased proportion of M2 macrophages and decreased proportion of M1 macrophages, may play crucial roles in the balance of pro-inflammatory and anti-inflammatory in the development of ACS. Furthermore, we found that heat shock protein genes were highly expressed but metal ion-related genes (S100 family and metallothionein family) were down-regulated in various subpopulations under high stress.

**Conclusions:**

We identified a high stress-specific subcluster and variations in immune cells and fibroblast subclusters, as well as their differentially expressed genes, in ACS patients. Our findings reveal the functions of the deep fascia in the pathophysiology of ACS, providing new approaches for its treatment and prevention.

## Introduction

Acute compartment syndrome (ACS), commonly caused by tibiofibular fractures (1), influences 3.1 per 100 000 people per year, with a strong male predominance of 7.3 per 100 000 men compared with 0.7 per 100 000 women (2). It is associated with local pain, paralysis, ischemia, or even necrosis of the lower limbs (3). These clinical symptoms are thought to be attributable to prolonged high stress in the compartment surrounded by the deep fascia (4). Our previous work demonstrated that scattered blisters surrounding fracture sections could significantly reduce the incidence of ACS. Additionally, inflammation has been reported as a major driving factor in the development of ACS (5, 6). Based on these findings, we hypothesize that the presence of blisters may relieve intracompartmental stress *via* immunologic mechanisms (7–9).

Fascia, broadly termed the “fascial system” (10, 11), is an uninterrupted, viscoelastic connective tissue that extends and interconnects between tissues and organs throughout the body (12). Fascia mainly consists of numerous fibers (13) that make it elastic to withstand tension (14), and fibroblasts that are involved in the production of collagen, tissue repair, and remodeling (15). Fibroblasts have been reported to contribute to the regulation of immune cells in the process of wound healing and scarring (16–19). Recent studies have mainly focused on the histochemical and mechanographic investigation on the lumbar or thoracolumbar fascia and found B cells, fasciacytes, myofibroblasts, telocytes, and fibroblasts (20–25). However, to our knowledge, the related research on deep fascia remains completely understood. We propose cellular subtypes, including fibroblasts and immune cells, gene expression, and signaling pathways in the deep fascia are inevitably altered under high stress in the compartment.

To verify these hypotheses, we applied single-cell RNA sequencing (scRNA-seq) to interrogate the cellular heterogeneity, gene expression, and cell-to-cell interactions of the deep fascia in patients with ACS. Our study identified subclusters of fibroblasts and immune cells from the deep fascia as exhibiting variations in response to high stress in the compartment, implying physiological specialization. To date, there is limited knowledge of the cellular heterogeneity of the deep fascia in ACS patients. We present molecular information to improve our understanding of the deep fascia and elucidate its functions in the development of ACS.

## Methods

### Patient recruitment and ethics

**Figure 1:**
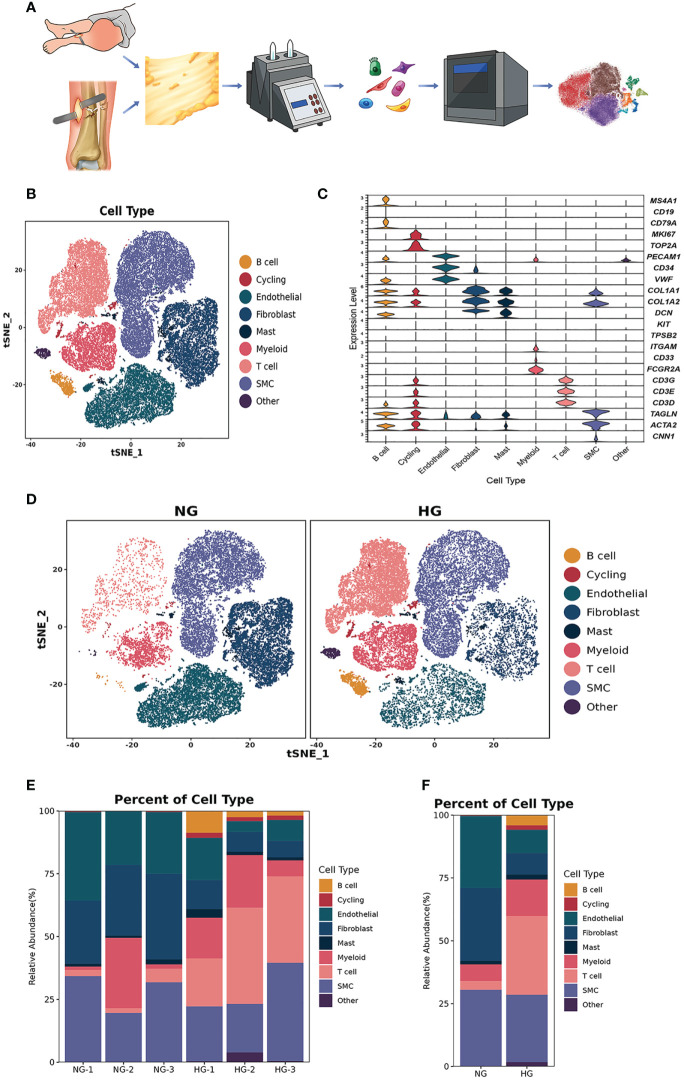
Clustering and classification of the cellular landscape of deep fascia tissue. **(A)** Overview of the experimental workflow. **(B)** tSNE visualization of cell cluster from deep fascia tissue. **(C)** Violin plots showing marker genes across cell clusters. **(D)** tSNE visualization of cell cluster from deep fascia tissue in HG and NG. **(E)** Bar plots showing the relative percentage of cell cluster for each sample as in **(A)**. **(F)** Bar plots showing the relative percentage of cell cluster in HG and NG. scRNA-seq, single-cell RNA sequencing; HG, high stress group; NG, normal stress group.

We collected deep fascia from patients with ACS after tibiofibular fractures, which were considered as the high-stress group (HG, n=3), and those with osteosarcoma who underwent thigh amputation, which were regarded as the normal-stress group (NG, n=3) in our hospital (**Figure 1A**). Due to a lack of fascia from healthy people, we selected patients receiving thigh amputations due to osteosarcoma who were not sensitive to targeted chemotherapy drugs to diminish the effect of osteosarcoma on the deep fascia. Basic patient information, such as age and sex, was recorded and presented in **Supplementary Table 1**. We obtained informed consent from six human subjects, and our study was approved by the Third Hospital of Hebei Medical University Ethics Committee (No. JS-1940).

### Sample preparation and tissue dissociation

A sterile RNase-free culture dish was placed on ice with PBS. The deep fascia samples were obtained from the medial shank of all participants. We cleaned blood spots and adipose tissue with 1×PBS and trimmed them into 0.5 mm^2^ fragments. Tissues were dissociated into single cells in a 37°C water bath with shaking at 100 rpm for 20 minutes in a dissociation solution (0.35% collagenase IV5, 2 mg/ml papain, 120 units/ml DNase I). Decomposition was stopped by pipetting 5-10 times with a Pasteur pipette in 1× PBS containing 10% fetal bovine serum (FBS, V/V). The cell suspension was then filtered using a 70-30 µm stacked cell strainer before being centrifuged at 300 × g for 5 minutes at 4°C. Single-cell suspensions were counted using a hemocytometer/Countess II Automated Cell Counter, and the concentration was adjusted to 700-1200 cells/μl. Overall cell viability was validated by trypan blue exclusion, which was required to be above 85%.

### Chromium 10x Genomics library and sequencing

According to the manufacturer’s instructions for the 10X Genomics Chromium Single-Cell 3’ Kit (V3), single-cell suspensions were placed into 10x Chromium to capture 8000 single cells. The processes for cDNA amplification and library creation were carried out according to the conventional procedure. LC-Bio Technology Co., Ltd. (Hangzhou, China) sequenced libraries using an Illumina NovaSeq 6000 sequencing system (paired-end multiplexing run, 150 bp) at a minimum depth of 20,000 reads per cell (**Figure 1A**).

### Single-cell RNA-seq data processing and data visualization

Illumina bcl2fastq software (version 2.20) was applied to demultiplex the sequencing data and converted it to FASTQ format. The Cell Ranger pipeline (version 6.1.1) was used to demultiplex samples, process barcodes, and count single-cell 3’ genes, and scRNA-seq data were aligned to the GRCh38 reference genome in Ensembl. A total of 64222 single cells were processed using 10X Genomics Chromium Single Cell 3’ Solution from three patients with blisters after tibiofibular fractures and three patients with thigh amputation (**Figure 1A**). The Cell Ranger output was loaded into Seurat (version 3.1.1) and used for dimensionality reduction, clustering, and analysis of scRNA-seq data. Then, to filter the environmental RNA contamination caused by these techniques, we used CellBender (version 0.2.0) to assess and adjust the results. Ultimately, 53116 cells passed the quality control threshold. These cells were kept if they met the following criteria: (1) they expressed genes in more than one cells; (2) they expressed more than 500 genes per cell; and (3) the percentage of mitochondrial DNA-derived gene expression was less than 25%. Doublets were removed using DoubletFinder.

We utilized Seurat to reduce the dimensionality of all 53116 cells and tSNE to project the cells into 2D space to display the data. The procedure is as follows: First, we calculated the expression value of genes using the LogNormalize technique of the Seurat software’s “Normalization” function; second, the normalized expression value was used to perform principal component analysis (R harmony), and the top 20 PCs were utilized for clustering and tSNE analysis; third, to locate clusters, the weighted Shared Nearest Neighbor graph-based clustering approach was applied. “bimod”: A likelihood-ratio test was used to find marker genes for each cluster using the FindAllMarkers function in Seurat. This method chooses marker genes that are expressed in more than 10% of the cells in a cluster and have an average log (fold change) larger than 0.26.

### Dimensionality reduction and annotation of major cell types

The number of unique molecular identifiers, percentage of mitochondrial genes, and genes were scaled to unit variance. Principal component analysis was performed. Clusters were then identified using tSNE. Cell identity was assigned using known markers.

### Detection of differentially expressed genes and pathway analysis

The differentially expressed genes (DEGs) between two groups comparing the same cell types were identified using the defalut parameters *via* the FindMarkers function in Seurat. These genes were categorized by average log2 (fold change) after being processed with a minimum log2 (fold change) of 0.26 and a maximum adjusted p value of 0.01. Gene Ontology (GO) terms and Kyoto Encyclopedia of Genes and Genomes (KEGG) pathways were used to create the gene sets.

### Cell-cell interaction analysis

CellPhoneDB (v3) was utilized to investigate cell-cell interactions between cells in depth. According to the concept of receptor expression by one cell subpopulation and ligand expression by another, we calculated possible ligand-receptor interactions. The normalized counts of cells in the two groups were independently downloaded and used as input for the CellPhoneDB method.

### Ultrasound

We performed an ultrasound to observe the differences in the morphology of the deep fascia between the HG and NG.

### Scanning electron microscopy

We observed changes in the morphology of the deep fascia in the HG and NG using scanning electron microscopy (HITACHI, SU8100). To avoid pulling and clamping of the fascia during sample selection, samples were collected within 1–3 minutes, washed in PBS (gently during washing), and subjected to the removal of surface impurities and blood stains. Then, a razor blade was used in an electron microscope fixator to repair blocks (while fixing blocks), and the tissue blocks were rectangular as often as possible, with a size of approximately 5 mm*3 mm. The surface was cut in the top right corner (to distinguish the surface). When collecting materials, we were careful to avoid mechanical damage, such as forceps extrusion, and the blade was sharpened to prevent tissue bruising. The scanning surface was handled with extreme care. The tissue was immediately fixed under an electron microscope after it was removed.

### Multiplex staining and multispectral imaging

The diverse cell subsets in the fascia were identified by multiplex staining and multispectral imaging using a PANO 5-plex IHC kit (cat 0004100100) (Panovue, Beijing, China). CD3, α-SMA, and CD14 were applied sequentially and incubated with a secondary antibody conjugated to horseradish peroxidase and a tyramide signal amplification reagent. Each slide was heat-treated using a microwave after TSA conjugation. DAPI was applied to label the location of nuclei after all the other antigens had been stained. To obtain high-quality images, all stained slides were scanned under the Mantra System (PerkinElmer, Waltham, Massachusetts, US). The fluorescence spectra were set at 20-nm wavelength intervals from 420 to 720 nm with identical exposure times.

## Results

### The landscape of various cells in HG and NG

**Figure 2:**
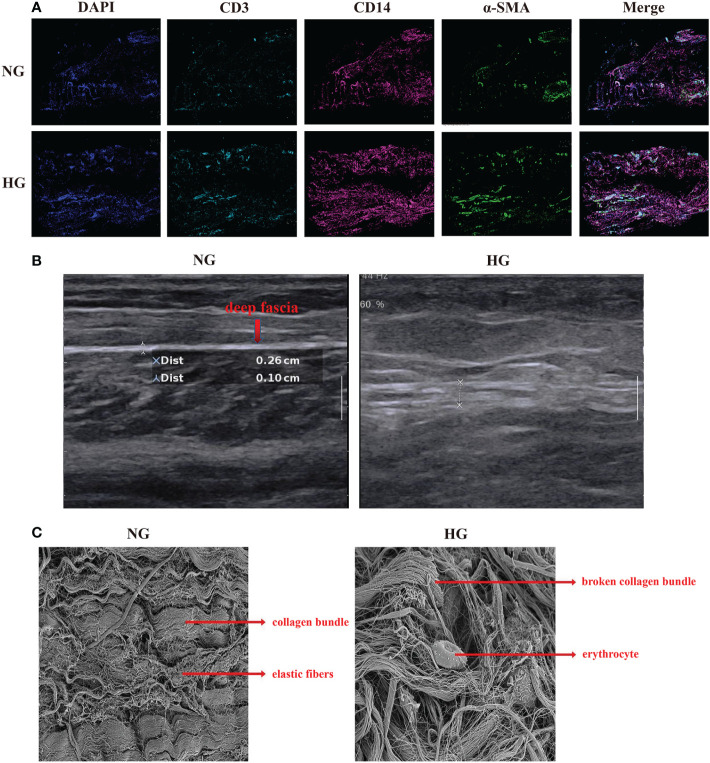
Multiplex staining and multispectral imaging, ultrasound and Scanning Electron Microscope for deep fascia in two groups. **(A)** Multiplex staining and multispectral imaging showing fibroblasts and immune cell infiltration, including T cells and myeloid cells in HG compared with NG. *green (α-SMA): fibroblast; pink (CD14): myeloid cells; blue (CD3): T cell. **(B)** ultrasound in patients with in HG (right side) compared with the NG (left side) in ultrasound. **(C)** Scanning Electron Microscope in HG and NG. Left: parallel arrangement of collagen fiber bundles in NG; right: chaotic overall arrangement of collagen fiber bundles in HG. HG, high stress group; NG, normal stress group.

After data preprocessing and quality control, we obtained single-cell transcriptomes of 53116 cells in two groups, including 27166 cells from the HG and 25950 cells from the NG. We applied unsupervised graph-based clustering to identify 9 clusters, which mainly consisted of B cells (*CD79, CD19*, and *MS4A1*), cycling cells (*TOP2A* and *MKI67*), endothelial cells (*VWF, PECAM1*, and *CD34*), fibroblasts (*COL1A1, COL1A2*, and *DCN*), mast cells (*KIT* and *TPSB2*), myeloid cells (*FCGR2A, CD33*, and *ITGAM*), T cells (*CD3G, CD3E*, and *CD3D*), smooth muscle cells (*CNN1, ACTA2*, and *TAGLN*), and other cells, by evaluating the expression of canonical markers (**Figures 1B–D**). **Figure 1E** showed the proportions of each cluster in different individuals, and we observed significantly higher proportions of B cells, T cells, cycling, mast cells, and myeloid cells but markedly lower proportions of fibroblasts, endothelial cells, and smooth muscle cells in the HG than in the NG (**Figure 1F**, **Supplementary Table 2**). This was consistent with the results of multiplex staining and multispectral imaging, which showed variations of fibroblasts and immune cells, including T cells and myeloid cells, in the HG compared with the NG (**Figure 2A**). Ultrasound showed a low-echo region surrounding the deep fascia in the HG but not in the NG (**Figure 2B**). Chaotic organization of collagen fiber bundles and broken collagen fibers could be observed in the HG compared with the NG (**Figure 2C**).

### Heterogeneous subpopulations of T-cell subsets in the two groups

**Figure 3:**
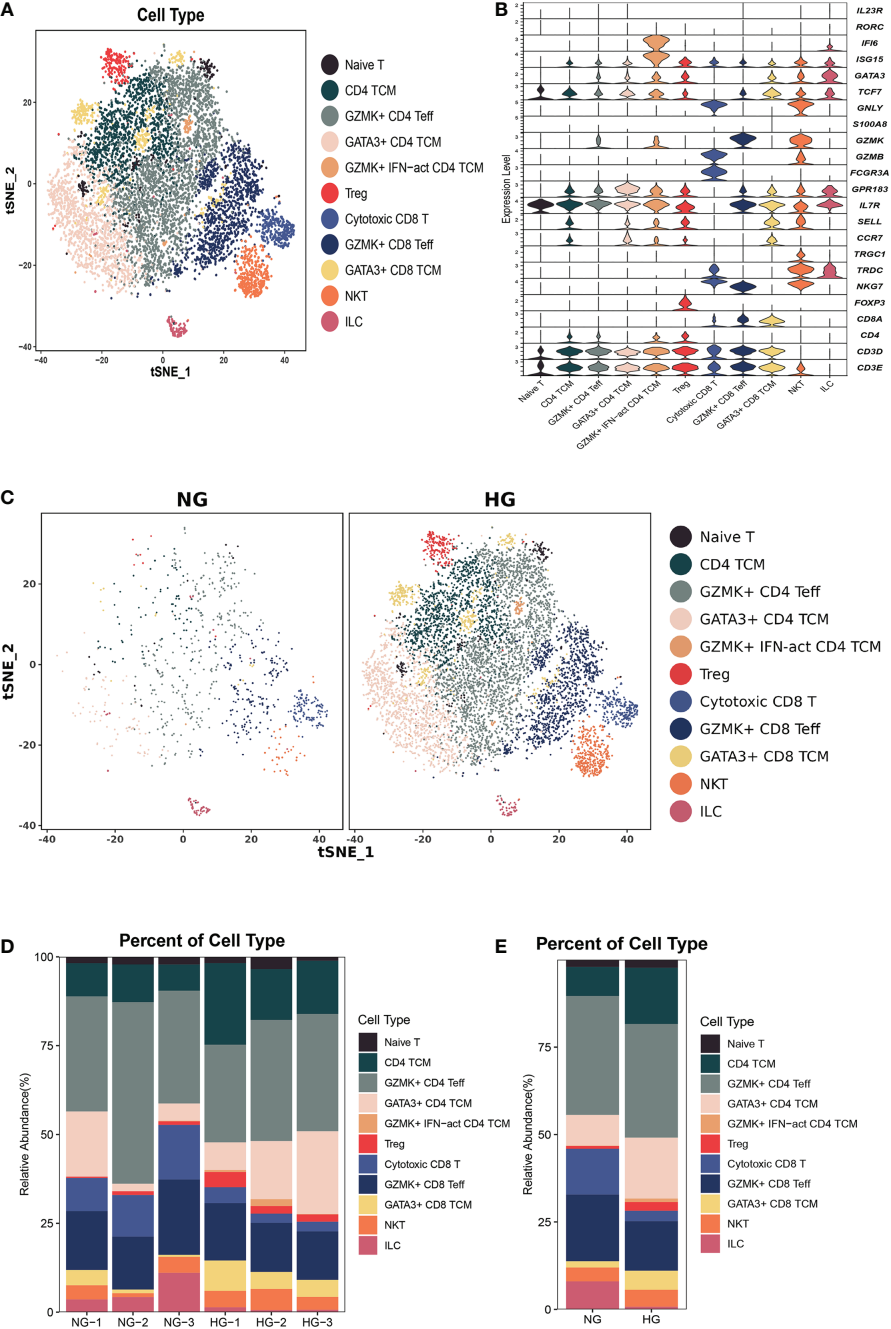
Clustering and classification of the T cell subclusters of deep fascia tissue. **(A)** tSNE visualization of T cell subclusters from deep fascia tissue. **(B)** Violin plots showing marker genes across T cell subclusters. **(C)** tSNE visualization of T cell subclusters from deep fascia tissue in HG and NG. **(D)** Bar plots showing the relative percentage of T cell subclusters for each sample. **(E)** Bar plots showing the relative percentage of T cell subclusters in HG and NG. HG, high stress group; NG, normal stress group; TCM, central memory T cell; Teff, effector T cell; Treg, regulator T cell; ILC=innate lymphoid cells; NKT=nature killer T cell.

To further explore the specific signatures of T cells in two groups, we subgrouped T cells into eleven cell subtypes: the Naive T-cell (*CD3D, CD3E, TCF7*, and *IL7R*), CD4 central memory T-cell (TCM; *CD4, TCF7, SELL, CCR7, GPR183, IL7R*), GZMK^+^ effector CD4 T-cell (Teff; *CD4, GZMK, GATA3*, and *ISG15*), GATA3^+^ CD4 TCM (*GATA3, TCF7, SELL, CCR7, GPR183, IL7R*), GZMK^+^interferon(INF)-act CD4 TCM (*IFI6, ISG15, CD4, TCF7, GZMK, GPR183, CCR7, SELL, IL7R*), regulatory T-cell (Treg; *FOXP3, CD4*), cytotoxic CD8 T-cell (*GNLY, FCGR3A, NKG7, TRDC*CD8A), GZMK^+^ CD8 Teff (*NKG7, GZMK, CD8A*), GATA3^+^ CD8 TCM (*GATA3, SELL, CCR7, IL7R, GPR183, TCF7, CD8A*), nature killer T-cell (NKT, *GNLY, TRDC, TRGC1* CD3D), and innate lymphoid cell (ILC, *RORC, IL23R, IF16*) subtypes (**Figures 3A–C**) (26). Next, we compared the proportions of T-cell subsets in the two groups. We observed higher proportions of CD4 TCM, GATA3^+^ CD4 TCM, GATA3^+^ CD8 TCM, GZMK^+^IFN-act CD4 TCM, and Treg cells, as well as lower proportions of cytotoxic CD8 T cells, GZMK^+^ CD8 Teff cells, and ILCs, in HG than in NG (**Figures 3C–E**, **Supplementary Table 2**). Notably, the GZMK^+^ IFN-act CD4 TCM subtype was almost absent in the NG but almost occurred in the HG.

**Figure 4:**
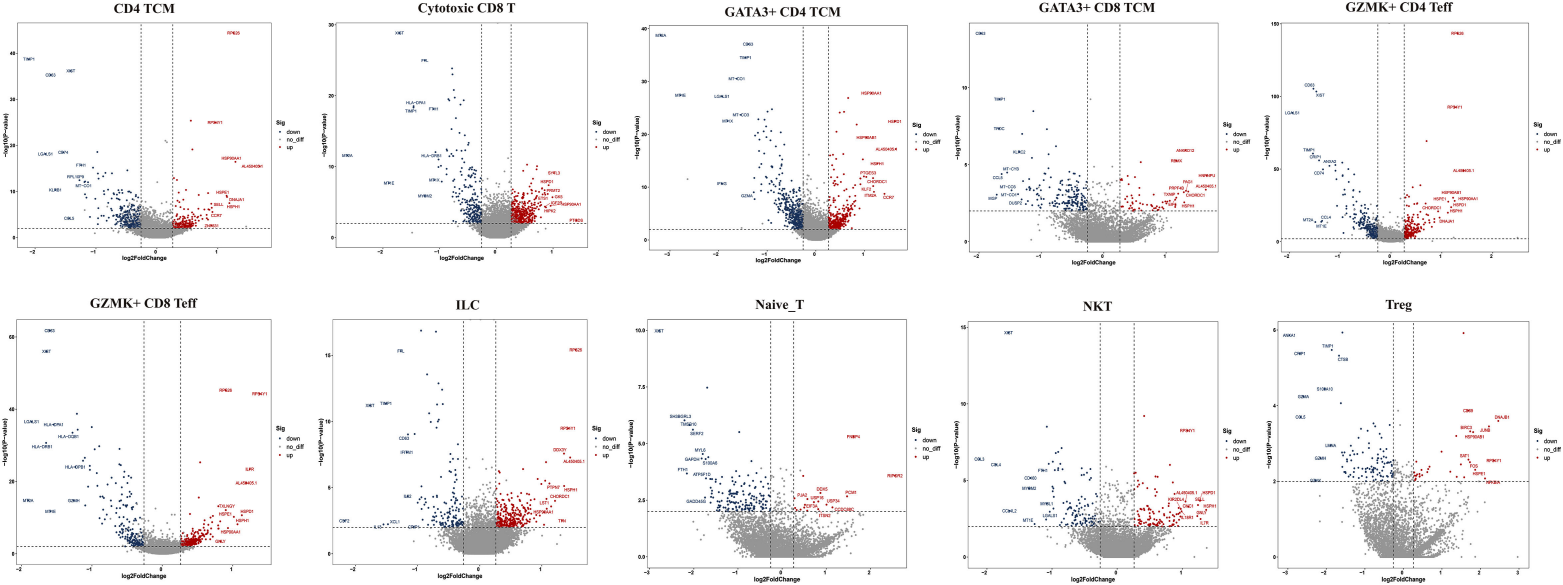
Top 10 differentially expressed genes (DEGs) of T cell subclusters. TCM, central memory T cell; Teff, effector T cell; Treg, regulator T cell; ILC, innate lymphoid cells; NKT, nature killer T cell.

Next, we compared the top 10 differentially expressed genes (DEGs) of T-cell subtypes in the two groups. In HG, heat shock protein (*HSP*) genes, such as *HSPH1* (*HSP110*), *HSP90AA1* (*HSP90*), *HSPA1A* (*HSP70*), *HSPA1B* (*HSP70*), *HSPD1* (*HSP60*), *DNAJA1* (*HSP40*), and *HSPE1* (*HSP10*), were up-regulated in nine subsets, except for GZMK^+^IFN-act CD4 TCM and naive T cells (**Figure 4**). As we know, *HSP* genes were associated with stress and the inflammatory response (27, 28). Additionally, metal ion-related genes, including metallothionein (*MT*) family genes (*MT2A*, *MT1E*, *MT1X*, *MT-CO1*, *MT-CO3*, and *MT-CYB*), *TIMP1*, *FTH1*, *FTL*, *CRIP1*, *MGP*, and *ANXA1* were down-regulated in these nine subclusters (**Figure 4**). In addition, we found the GZMK^+^IFN-act CD4 TCM subset nearly in the HG, with high expression of IFN-related genes (*ISG15*, *IFI6*), *GATA3*, and *GZMK* (**Figures 3B–E**). We also found Treg had a higher expression of *CD69* and GATA3^+^CD4 TCM had a higher expression of *KLF2* in HG.

Then, we performed GO and KEGG pathway analyses in the two groups. The CD4 TCM, cytotoxic CD8 T, GATA3^+^CD4 TCM, GATA3^+^CD8 TCM, and GZMK^+^CD4 Teff subtypes were mainly involved in “extracellular exosome”, “antigen processing and presentation” and “oxidative phosphorylation“ (**Supplementary Figures 1**, **2**). Cytotoxic CD8 T, GATA3^+^CD4 TCM, GATA3^+^CD8 TCM, and GZMK^+^CD4 Teff cells were mostly engaged in “Th1, Th2, and Th17-cell differentiation” (**Supplementary Figures 1**, **2**). GZMK^+^CD8 Teffs and GZMK^+^CD4 Teffs were related to the “MHC class II protein complex” (**Supplementary Figures 1**, **2**). The GZMK^+^IFN-act CD4 TCM subtype was linked to “response to type I interferon, interferon-gamma, interferon-beta, and interferon-alpha”. Moreover, some subpopulations played crucial roles in signaling pathways. For example, the GATA3^+^CD8 TCM was related to “HIF signaling pathway”, NKT cells were involved in “Chemokine signaling pathway” and “CCR chemokine receptor binding”, Treg cells were associated with “IL-17 signaling pathway”, “NF-kappa B signaling pathway”, “TNF signaling pathway”, and “Ferroptosis”, and the GZMK^+^IFN-act CD4 TCM was linked to “NF-kappa B signaling pathway”, “type I interferon signaling pathway”, “TNF mediated signaling pathway” and “interferon-gamma mediated signaling pathway” (**Supplementary Figures 1**, **2**).

Overall, we identified a specific subcluster in the HG, GZMK^+^IFN-act CD4 TCM, with high expression of IFN-related genes, which was almost absent in the NG but was almost present in the HG. Furthermore, we found that *HSP* genes were highly expressed in nine of eleven T-cell subclusters under high stress. Additionally, some signaling pathways may be important regulators of molecular mechanisms under high stress in the deep fascia.

### Heterogeneous subpopulations of macrophage subsets in the two groups

**Figure 5:**
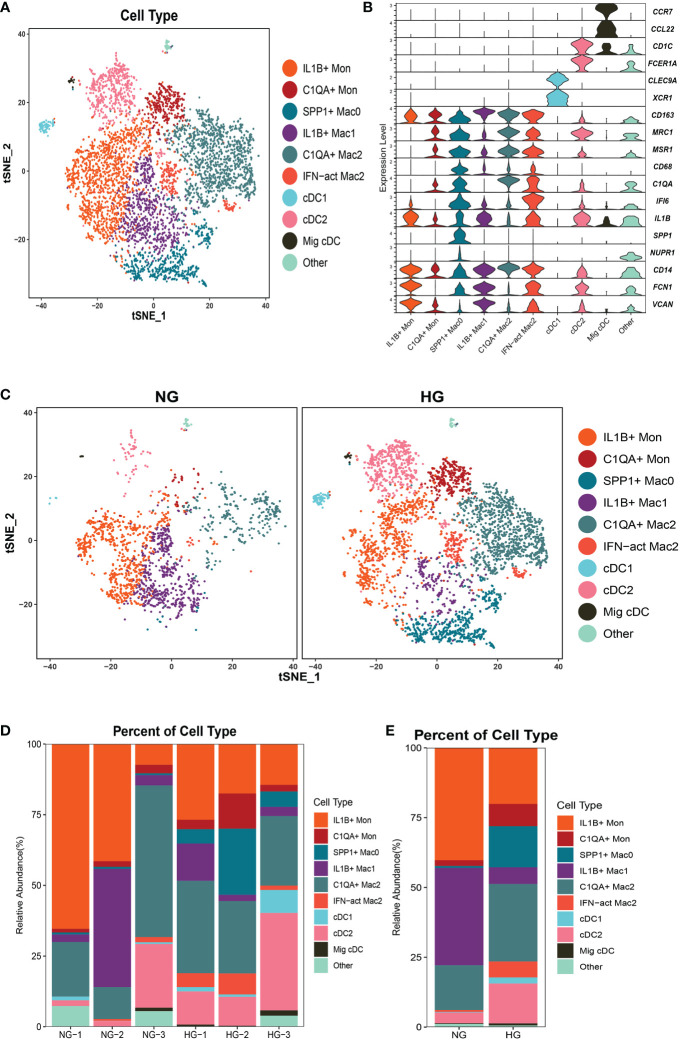
Clustering and classification of the myeloid cell subclusters from deep fascia tissue **(A)** tSNE visualization of myeloid cell subclusters from deep fascia tissue. **(B)** Violin plots showing marker genes across myeloid cell subclusters. **(C)** tSNE visualization of myeloid cell subclusters from deep fascia tissue in HG and NG. **(D)** Bar plots showing the relative percentage of myeloid cell subclusters for each sample. **(E)** Bar plots showing the relative percentage of myeloid cell subclusters in HG and NG. HG, high stress group; NG, normal stress group; Mac, macrophage; DCs, dendritic cells; Mon, monocyte.

**Figure 6:**
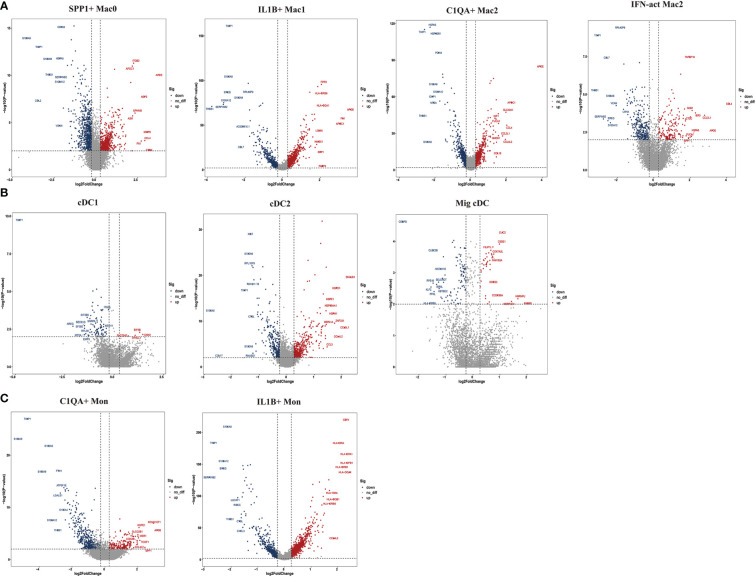
Top 10 differentially expressed genes (DEGs) of myeloid cell subclusters in HG and NG. **(A)** DEGs of macrophage (Mac) subclusters in HG and NG. **(B)** DEGs of dendritic cells (DCs) subclusters in HG and NG. **(C)** DEGs of monocyte (Mon) subclusters in HG and NG. HG, high stress group; NG, normal stress group.

To further explore the specific signatures of macrophages (Mac) in the two groups, we identified four subclusters: SPP1^+^Mac0 (*SPP1*, *VCAN, NUPR1*), IL1B^+^ Mac1 (*VCAN, FCN1, CD14, IL1B, IF16, CD68*), C1QA^+^ Mac2 (*VCAN, C1QA, CD68, CD163*) and IFN-act Mac2 (*IF16, C1QA, CD68, CD163*) (26, 29) (**Figures 5A–C**). We observed higher proportions of SPP1^+^ Mac0, C1QA^+^ Mac2, and IFN-act Mac2, as well as lower proportions of IL1B^+^ Mac1, in HG than in NG (**Figures 5D, E**; **Supplementary Table 2**). In terms of the top 10 DEGs, *APOE* was up-regulated, but the *S100* family (*S100A6, S100A8, S100A9, S100A10*, or *S100A12*) was down-regulated in four subclusters (**Figure 6A**). Additionally, *SPP1* was up-regulated in SPP1^+^Mac0 and IL1B^+^Mac1 under high stress, while *APOC1* was up-regulated in SPP1^+^Mac0.

The GO term and KEGG pathway data showed that SPP1^+^ Mac0 was involved in “antigen processing and presentation” and “oxidative phosphorylation”. IL1B^+^Mac1 participated in “antigen processing and presentation”, “oxidative phosphorylation”, “NF-kappa B signaling pathway”, and “ferroptosis”. C1QA^+^Mac2 was involved in the “cytokine-mediated signaling pathway”, “NF-kappa B signaling pathway”, and “ferroptosis”. IFN-act Mac2 was related to “inflammatory response”, “cellular response to interferon-gamma”, and “NOD-like receptor signaling pathway” (**Supplementary Figures 3**, **4**).

Taken together, these data revealed significant variation in the proportions of four Mac subclusters from deep fascia under high stress. Furthermore, a few signaling pathways may be activated in molecular mechanisms following fascia injury.

### Heterogeneous subpopulations of dendritic cell subsets in the two groups

To further explore the specific signatures of dendritic cells (DCs) in the two groups, we identified three DC subtypes (cDC1s (*XCR1, CLEC9A*), cDC2s (*FCN1, CD14, IL1B, C1QA, MSR1, MRC1, CD163, FCER1A, CD1C*), and Migratory cDCs (Mig cDCs*, IL1B, CD1C, CCL12, CCR7*)) (26, 29), and all three DC subtypes had significantly higher proportions in HG (**Figures 5A–E**; **Supplementary Table 2**). In terms of the top 10 DEGs, *HSP* genes (*HSPA1A, HSP90AA1, HSPD1*, and *HSPH1*) and chemokine genes (*CCL3L1* and *CCL4L2*) were up-regulated in cDC2s under high stress. Notably, metal ion-related genes were down-regulated in three DC subpopulations in HG. For example, *TIMP1 and MT2A* were down-regulated in cDC1s*. TIMP1, S100A6, S100A8, and S100A9* were down-regulated in cDC2s, as well as *PFKL* in Mig cDCs (**Figure 6B**). The GO term and KEGG pathway data showed that cDC1 cells were associated with the “homeostasis process”, as well as the HIF signaling pathway, p53 signaling pathway, and Notch signaling pathway. cDC2s were involved in the “inflammatory response” and “extracellular exosome”, as well as the IL-17 signaling pathway, chemokine signaling pathway, and NF-kappa B signaling pathway. Mig cDCs participated in the FoxO signaling pathway (**Supplementary Figures 5**, **6**).

Taken together, these data revealed significant variations in the proportions of three cDC subclusters in the deep fascia under high stress. Additionally, some signaling pathways may be engaged in molecular mechanisms after fascia injury.

### Heterogeneous subpopulations of monocyte subsets in the two groups

To further explore the specific signatures of monocytes (Mon) in the two groups, we identified two Mon subtypes C1QA^+^ Mon (*VCAN, CD14, IL1B, C1QA, MSR1, MRC1, CD163*) and IL1B^+^ Mon (*VCAN, FCN1, CD14, IL1B, IF16, CD163*)) (26, 29) and found higher proportions of C1QA^+^ Mon and lower proportions of IL1B^+^ Mon in HG than in NG (**Figure 5A–E**; **Supplementary Table 2**). Regarding the two Mon subclusters, *CCL4L2* was up-regulated, while S100 family members, *TIMP1* and *THBS1* were down-regulated in HG (**Figure 6C**). Furthermore, HLA family members (*HLA-DRA, HLA-DPB1, HLA-DQA1, HLA-DPA1, HLA-DRB1*, and *HLA-DRB6*) were up-regulated in IL1B^+^ Mon in HG (**Figure 6C**). The data for GO terms and KEGG pathways showed that IL1B^+^ Mon was involved in “inflammatory response”, “extracellular exosome”, and “NOD-like receptor signaling pathway” (**Supplementary Figure 7**).

Taken together, these data revealed significant variation in the proportions of two Mon subclusters in deep fascia under high stress. Furthermore, a few signaling pathways may be activated in molecular mechanisms following fascia injury.

### Heterogeneous subpopulations of fibroblast subsets in the two groups

**Figure 7:**
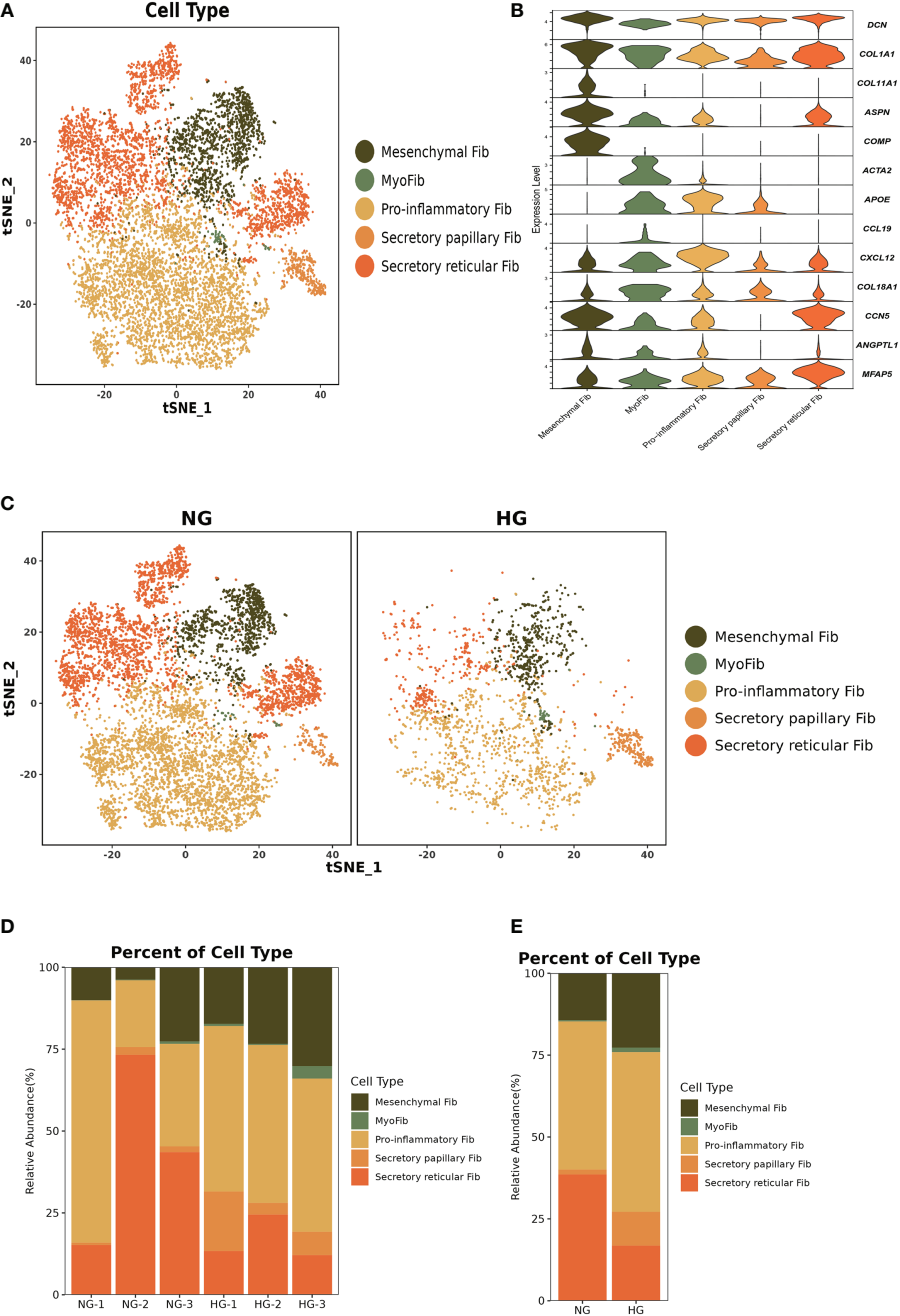
Clustering and classification of the fibroblasts (Fib) subclusters of deep fascia tissue **(A)** tSNE visualization of fibroblasts subclusters from deep fascia tissue. **(B)** Violin plots showing marker genes across fibroblasts subclusters. **(C)** tSNE visualization of fibroblasts subclusters from deep fascia tissue in HG and NG. **(D)** Bar plots showing the relative percentage of fibroblasts subclusters for each sample. **(E)** Bar plots showing the relative percentage of fibroblasts subclusters in HG and NG. HG, high stress group; NG, normal stress group.

**Figure 8:**
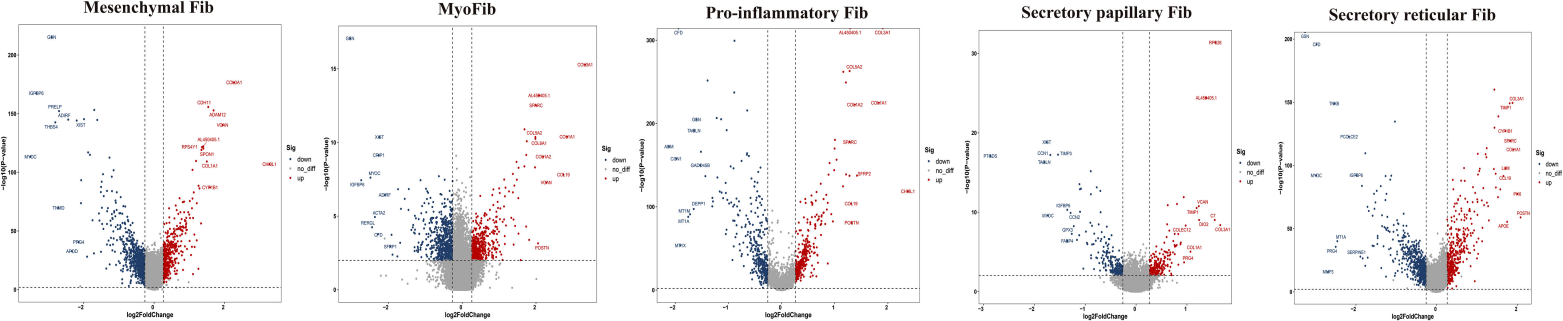
Top 10 differentially expressed genes (DEGs) of fibroblasts (Fib) subclusters in HG and NG. HG, high stress group; NG, normal stress group.

To further explore the specific signatures of fibroblasts (Fib) in the two groups, we subdivided fibroblasts into five cell subtypes: the mesenchymal Fib (*POSTN, COMP, ASPN*), myoFib (*RGS5, ACTA2*), proinflammatory Fib (*CCL19, CXCL12, APOE*), secretory papillary Fib (*COL13A1, COL18A1, APCDD1*) and secretory reticular Fib (*MFAP5, ANGPTL1, CCN5*) subtypes (30) (**Figures 7A–C**). Next, we compared the proportions of fibroblast subsets in the two groups. Interestingly, the proportions of the mesenchymal Fib, myoFib, pro-inflammatory Fib, and secretory papillary Fib subtypes dramatically increased, and a lower proportion of the secretory reticular Fib subtype was observed in the HG (**Figures 7D, E**, **Supplementary Table 2**). Next, we compared the top 10 DEGs of fibroblasts in the two groups. Regarding all fibroblast subclusters, fibrillar collagen genes, such as *COL1A1 and COL3A1* were up-regulated in HG (**Figure 8**). Notably, *COL1A2* and *COL5A2* were up-regulated in myoFib and pro-inflammatory Fib (**Figure 8**). Additionally, *POSTN* and *SPARC* were up-regulated in myoFib, pro-inflammatory Fib, and secretory reticular Fib. Genes that were engaged in metal ion binding, such as *MYOC, GSN, and IGFBP6*, were down-regulated in four subclusters (**Figure 8**). The GO term data showed that the five subpopulations were involved in “collagen fibril organization”, “collagen-containing extracellular matrix”, and “extracellular space” (**Supplementary Figure 8**). KEGG pathway analysis showed that the PI3K-Akt signaling pathway and TGF-beta signaling pathway may be involved in the regulation of pressure in the compartment (**Supplementary Figure 9**). Additionally, the mesenchymal Fib, myoFib, proinflammatory Fib, and secretory papillary Fib subtypes were engaged in “protein digestion and absorption”.

Taken together, these data revealed significant variation in the proportions of five Fib subclusters in the deep fascia under high stress. Additionally, some signaling pathways may be engaged in molecular mechanisms after fascia injury.

### Intercellular crosstalk among fibroblasts, myeloid cell subsets and T-cell subclusters

**Figure 9:**
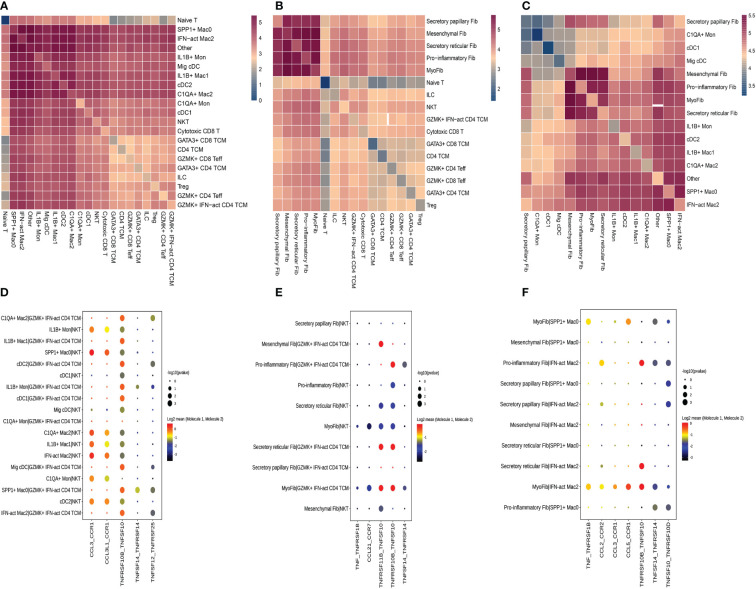
Crosstalk among T cell, fibroblasts and myeloid cell. **(A)** Heatmap presents the key cellular interaction between T cell and myeloid cell. **(B)** Heatmap presents the key cellular interaction between T cell and fibroblasts. **(C)** Heatmap presents the key cellular interaction between fibroblasts and myeloid cell. **(D)** Dot plot shows the significant ligand-receptor pairs between T cell subsubsets and myeloid cell subsubsets. **(E)** Dot plot shows the significant ligand-receptor pairs between T cell subsubsets and fibroblasts subsubsets. **(F)** Dot plot shows the significant ligand-receptor pairs between myeloid cell subsubsets and fibroblasts subsubsets.

We used CellPhoneDB to evaluate the interactions among fibroblast subclusters, myeloid cell subclusters, and T-cell subclusters. We found that each subcluster exhibited interactions with other subsets (**Figures 9A–C**). Then, a series of immune-related ligand-receptor pairs were applied to investigate the L-R pairs involved in these cell-cell interactions. GZMK+IFN-act CD4 TCM subsets mainly interacted with myeloid cell subsets *via* TNFRSF10B-TNFSF10 and TNFSF12-TNFRSF25 (**Figure 9D**), while NKT were more likely to use CCL3-CCR1, CCL3L1-CCR1, and TNFRSF10B-TNFSF10 (**Figure 9D**). GZMK+IFN-act CD4 TCM and NKT interacted primarily with fibroblast subsets *via* the TNFRSF10B-TNFSF10 and TNFSF11B-TNFRSF10 pathways (**Figure 9E**). Notably, myofib interacted with IFN-act Mac2 *via* TNF-TNFRSF1B, CCL2-CCR2, CCL3-CCR1, CCL5-CCR1, TNFRSF10B-TNFSF10, TNFSF14-TNFRSF14, and TNFSF10-TNFRSF10D, while myofib interacted with SPP1+ Mac0 *via* TNF-TNFRSF10D. Proinflammatory Fib interacted with IFN-act Mac2 *via* CCL2-CCR2, TNFRSF10B-TNFSF10, TNFSF14-TNFRSF14, and TNFSF10-TNFRSF10D (**Figure 9F**).

## Discussion

ACS, one of the devastating complications after tibiofibular fractures, is caused by high stress in the compartment surrounded by deep fascia (31). ACS may cause some serious complications, such as ischemia of the contained tissues, muscle necrosis, neurologic deficits, or even necrosis (31–34). Given the limitations of surgical treatment, effective new therapies for ACS are urgently required. Traditional evidence considers deep fascia as the fibrous connective tissue surrounding the skeletal muscles (35), yet recent ongoing research has focused on physiological and metabolic homeostasis, as well as healing and repair mechanisms (20–22). Prior research indicated that the stress response can alter the fascia and related tissues (36). To our knowledge, no study has used scRNA-seq to investigate the relationship between the stress response and deep fascia. To further investigate how the stress response affects the immune system of the deep fascia, we first utilized scRNA-seq to explore the cellular heterogeneity and molecular mechanism of the deep fascia in patients with ACS, which can help us better recognize the deep fascia and may provide more effective therapeutic strategies for ACS.

It is well known that the pressure in the compartment grows along with the swelling of muscles after injury. Once the pressure rises to some level, the surrounding tissues reach the hypoxic and ischemic microenvironment, resulting in aseptic inflammation (37–40). Our findings showed that various T cell subclusters infiltration under high stress, and the proportions of CD4 TCM, GATA3+CD4 TCM, GATA3+CD8 TCM, GZMK+IFN-act CD4 TCM, and Treg significantly increased in HG. This constellation of abnormally distributed immune cells suggests that the local response was substantially distinct under high stress. The proportion of Tregs rose from 0.79% in NG to 2.48% in HG, and they highly expressed the level of *CD69*. As we know, CD69+Tregs play a critical role in the maintenance of immunologic tolerance and suppression of innate immune responses (41). We also found higher proportions of two subclusters (CD4 TCM and CD8 TCM) with high expression of *GATA3* that contributed to the maintenance of immune activity (42) under high stress. Moreover, GATA3+CD4 TCM with a high expression of *KLF2* that was associated with the production of Treg (43) implied that GATA3+CD4 TCM may be a regulator of Treg.

Furthermore, we found that *HSP* genes, including *HSP110, HSP90, HSP70*, *HSP60*, and *HSP10*, highly expressed in nine of the eleven T cell subsets, in addition to GZMK+IFN-act CD4 TCM and Naive T cell. *HSP* genes are known to respond to different stressors such as ischemia and hypoxia (44) and their functions are involved in protection against apoptotic exchanges (45) and suppression of proinflammatory cytokines (46). Recent evidence has demonstrated their protective roles in oxidative stress and ischemia/reperfusion injury of some organs, such as the liver (47), brain (48), spinal cord (49), and heart (50). T cells that react to self-HSPs and act as suppressor cells have subsequently been described, and they are regarded as playing critical roles in maintaining peripheral tolerance or suppressing inflammation in animal models of rheumatoid arthritis, type 1 diabetes mellitus, or atherosclerosis (51–54). Some studies have found that children with HSP60-reactive T cells in their peripheral blood have a better prognosis than those who do not, and that a lack of HSP-responsive Tregs is linked to a variety of autoimmune disorders (55, 56). According to Kim et al. (57), the renoprotective effect of HSP70 may be partially mediated by a direct immunomodulatory action *via* Tregs. These nine subpopulations of T cells, especially Tregs, may be involved in protecting apoptosis and suppressing proinflammatory cytokines under high stress, which provides a novel insight into the treatment of ACS. In contrast, the *MT* family, which was found to have a role in the reduction of oxidative damage and apoptosis in animal models and was involved in the binding of a large number of heavy metal ions such as zinc ions, copper ions, or cadmium ions (58, 59), was down-regulated in nine subsets (60–62). We suppose that *HSP*, *MT*, or the ratio of *HSP* to *MT* may be an important disease biomarker in the diagnosis of ACS, or maybe a potential key factor of ACS that is worthy of study at the protein level.

Another important finding in our study was a rare T cell subpopulation, GZMK+IFN-act CD4 TCM, which was almost exclusively identified in HG. It highly expressed INF-related genes, including *ISG15* and *IFI6*. *ISG15* has been reported to rarely express under normal conditions (63, 64), and their potential as biomarkers, drug targets, or immunotherapy choices for some diseases because they are substantially up-regulated in cancer (65), neurodegenerative diseases (66), or inflammatory disease (67). Similar to these findings, upregulation of *ISG15* in the present study in HG suggested that it may be a suitable biomarker for detecting pressure variation in the compartment. *GATA3*, highly expressed in GZMK+IFN-act CD4 TCM, played a crucial role in regulating the homeostatic survival, proliferation, and differentiation of T-cell subsets (68, 69), suggesting that GZMK+IFN-act CD4 TCM may be a key regulator in the development of T cells. *GZMK*, a pro-inflammatory granzyme family member, was previously thought to be cytotoxic proteases, but recent research found that *GZMK* could regulate the inflammatory response rather than inducing cell death (70), implying that GZMK^+^IFN-act CD4 TCM may play an important role in the regulation of the inflammatory response. However, it still needs to be further investigated. Taken together, our findings first identified a specific-disease T cell subcluster with high expression of *ISG15, IFI6, GATA3*, and *GZMK*. Additionally, up-regulation of *HSP* genes and down-regulation of *MT* genes in T cell subclusters were found in HG. These findings might facilitate the discovery of new preventive strategies for high-pressure-derived diseases of the deep fascia, such as ACS.

The S100 proteins are evolved in multiple cellular processes such as cell apoptosis, proliferation, differentiation, migration, energy metabolism, calcium balance, protein phosphorylation, and inflammation (71, 72), as well as contributing to the development of various diseases, such as autoimmune diseases (73) and chronic inflammatory disorders (74). They have been used as diagnostic markers to aid in differential diagnosis (75) and therapeutic targets in some diseases (76). Down-regulation of *S100A8* and *S100A9* was shown to inhibit the differentiation of myeloid cells toward DCs and Mac (pro-inflammatory M1 or anti-inflammatory M2) (77). In the current study, the *S100* family was down-regulated in two Mon subclusters (C1QA^+^Mon, IL1B^+^Mon) and four Mac subpopulations (SPP1^+^Mac0, IL1B^+^Mac1, C1QA^+^Mac2, and IFN-act Mac2). M1 macrophages are related to pro-inflammatory factors and M2 macrophages are linked to anti-inflammatory factors (78). Notably, our findings showed a decreased proportion of M1 cells and an increased proportion of M2 cells under high stress, which was consistent with other evidence of anti-inflammatory effects that decreased the expression of chemokine genes in six subsets of T cells. We also found SPP1^+^Mac0 with high expression of *APOC1* which promoted M2 polarization of macrophages (79), indicating that SPP1^+^Mac0 was involved in the polarization of macrophages. We infer that the balance of M1 and M2 macrophages may play a crucial role in the development of ACS and even maybe a therapeutic target for ACS. We also found that *APOE* which was associated with lipid homeostasis and muscle regeneration according to previous studies was up-regulated in four Mac subclusters (80, 81), implying that Mac may play an important role in muscle injury for patients with ACS. Additionally, *SPP1* which has been reported to be related to fibrosis in renal cardiac and bone marrow was up-regulated in SPP1^+^Mac0 and IL1B^+^Mac1 (82–85), suggesting that these subclusters may have a general role in promoting fibrosis in patients with ACS. Our future study will observe the dynamics of the immune system with pressure alteration within the compartment. The lack of widely accepted animal models limits our investigation of the deep fascia under high stress, so there is an urgent need to establish an animal model to mimic the development of ACS.

Fibroblasts, a major cluster of deep fascia, have different secretory capabilities due to highly heterogeneous and distinct organs (86–89). Fibroblast heterogeneity and function have been well described in multiple fibrotic diseases, such as lung fibrosis, systemic sclerosis, Dupuytren’s disease, and keloid (90–92). However, to our knowledge, a study of scRNA-seq application for exploring fibroblast heterogeneity in deep fascia under high pressure is still absent. Our findings showed dramatically lower proportions of fibroblast subsets in HG, which may be associated with immune cell infiltration. We found proportions of mesenchymal Fib, myoFib, pro-inflammatory Fib, and secretory papillary Fib dramatically increased and a lower proportion of secretory reticular Fib under high stress. Interestingly, we found the proportions of secretory papillary Fib rose 5.88-fold under high stress in the compartment. Five subclusters highly expressed fibrillar collagen genes, such as *COL1A1* and *COL3A1*, which were involved in the synthesis and repair of collagen that mainly contributed to the structural integrity of extracellular matrix, tissues, and organs (93, 94). Traction force generated by myoFib has been reported to greatly contribute to collagen formation and modulates the activities of stromal cells after injury (95, 96), which was consistent with our findings that myoFib in patients with ACS highly expressed *COL1A2, COL5A2, POSTN*, and *SPARC* that were associated with fibrosis (97, 98). Additionally, pro-inflammatory Fib and secretory reticular Fib in HG also expressed these genes, implying that these subclusters may be involved in the fibrosis of deep fascia after injury. Five subpopulations were involved in the “extracellular space”, indicating a change in the extracellular space in the deep fascia in response to high stress. Regarding the skin, the papillary Fib subtype contributes to the attachment between the epidermis and the dermis to maintain skin elasticity, while the reticular Fib subtype produces large amounts of collagen and elastin fibers (99). We can infer that the ratio of reticular Fib to papillary Fib was related to elasticity, which may determine the occurrence of ACS.

We concentrated on the higher proportions of subclusters in HG or on these subclusters that interacted more with others due to a large amount of data on cell-cell interactions. GZMK+IFN-act CD4 TCM subsets mainly interacted with myeloid cell subsets or fibroblast subsets *via* the TNF-TNFRSF axis, while NKT were more likely to use the CCL-CCR axis and the TNF-TNFRSF axis. Regarding the cell-cell interactions between fibroblast subsets and macrophage subclusters, they are mainly mediated by the CCL-CCR axis and the TNF-TNFRSF axis. These results indicated that there were complicated relationships among these distinct cell types and described the cellular crosstalk network that regulates the homeostasis of the deep fascia after injury. Previous studies have reported the role of fibroblasts in immunological functions by influencing the differentiation, movement, and activation of immune cells (16, 100, 101), which is consistent with our findings that fibroblasts closely interact with immune cells *via* chemokines and cytokines. Given the lack of a widely accepted animal model for ACS, we failed to directly validate our findings in an animal model, including potential functions and interactions between immune cells and fibroblast populations. Meanwhile, we were unable to perform cellular experiments due to a lack of fascia-derived fibroblasts.

There are still some limitations. First, dynamic variations in immune cells and fibroblasts cannot be observed due to the small size of the samples. Second, individual differences may have biased the interpretation of the results. Additionally, the lack of deep fascia from healthy patients may impact the accuracy of results. Third, we failed to sort out subclusters of major cells, such as B cells, cycling cells, endothelial cells or smooth muscle cells according to biomarkers from published studies. In this study, we paid more attention to variations of fibroblasts and immune subclusters in patients with ACS. However, to our knowledge, this is the first study to identify the variations in cellular heterogeneity and the molecular mechanism of the deep fascia in patients with ACS by the scRNA-seq technique.

## Conclusions

Our study provides the first landscape of cellular subclusters from the deep fascia in patients with ACS at the single-cell level. We identified variations in immune cells and fibroblast subclusters, as well as their differentially expressed genes. Notably, we identified a GZMK^+^IFN-act CD4 TCM subtype expressing IFN-related genes in ACS patients. Furthermore, the variations in M1/M2 polarization and the ratio of reticular Fib to papillary Fib may play crucial roles in the development of ACS. Additionally, *HSP* genes were up-regulated in various T-cell subclusters, and *S100* family genes and *MT* family genes were down-regulated in numerous immune cell subclusters under high stress. Our work provides a solid foundation and offers novel insights for further research on the deep fascia, which helps us recognize the function of the deep fascia and may provide effective treatments for ACS.

## Data availability statement

The data presented in the study are deposited in the The National Center for Biotechnology Information repository, accession number PRJNA890689.

## Ethics statement

The study was approved by the Institutional Review Board of the third hospital of Hebei Medical University before data collection and analysis. The patients/participants provided their written informed consent to participate in this study. Written informed consent was obtained from the individual(s) for the publication of any potentially identifiable images or data included in this article.

## Author contributions

Conceptualization: TW; data curation: TW and YBL; formal analysis: LJM; Investigation: QD and YRL; methodology: JFG; Project Administration:LJ; Resources: TW and YBL; Software: QD; Supervision: YZZ; Visualization: LQD; Writing-original draft: TW writing-review and editing: ZYH and LW. All authors contributed to the article and approved the submitted version.

## Supplementary material

Supplementary Figure 1 Gene Ontology (GO) terms of T cell subtypes. (TCM=central memory T cell; Teff=effector T cell; Treg=regulator T cell; ILC=innate lymphoid cells; NKT=nature killer T cell)

Supplementary Figure 2 Kyoto Encyclopedia of Genes and Genomes (KEGG) pathways of T cell subtypes. (TCM=central memory T cell; Teff=effector T cell; Treg=regulator T cell; ILC=innate lymphoid cells; NKT=nature killer T cell)

Supplementary Figure 3 Gene Ontology (GO) terms of macrophage (Mac) subtypes.

Supplementary Figure 4 Kyoto Encyclopedia of Genes and Genomes (KEGG) pathways of macrophage (Mac) subtypes.

Supplementary Figure 5 Gene Ontology (GO) terms of dendritic cells (DCs) subtypes.

Supplementary Figure 6 Kyoto Encyclopedia of Genes and Genomes (KEGG) pathways of dendritic cells (DCs) subtypes.

Supplementary Figure 7 Gene Ontology (GO) terms and Kyoto Encyclopedia of Genes and Genomes (KEGG) pathways of monocyte (Mon) subtypes. **(A)** GO terms of Mon subtypes. **(B)** KEGG pathways of Mon subtypes.

Supplementary Figure 8 Gene Ontology (GO) terms of fibroblast (Fib) subtypes.

Supplementary Figure 9 Kyoto Encyclopedia of Genes and Genomes (KEGG) pathways of fibroblast (Fib) subtypes.
