# Supplementary figures and images for: Single-cell RNA-seq reveals cellular heterogeneity from deep fascia in patients with acute compartment syndrome
Source: Front Immunol. 2023 Jan 18;13:1062479. doi: 10.3389/fimmu.2022.1062479 (PMC9889980; doi:10.3389/fimmu.2022.1062479)

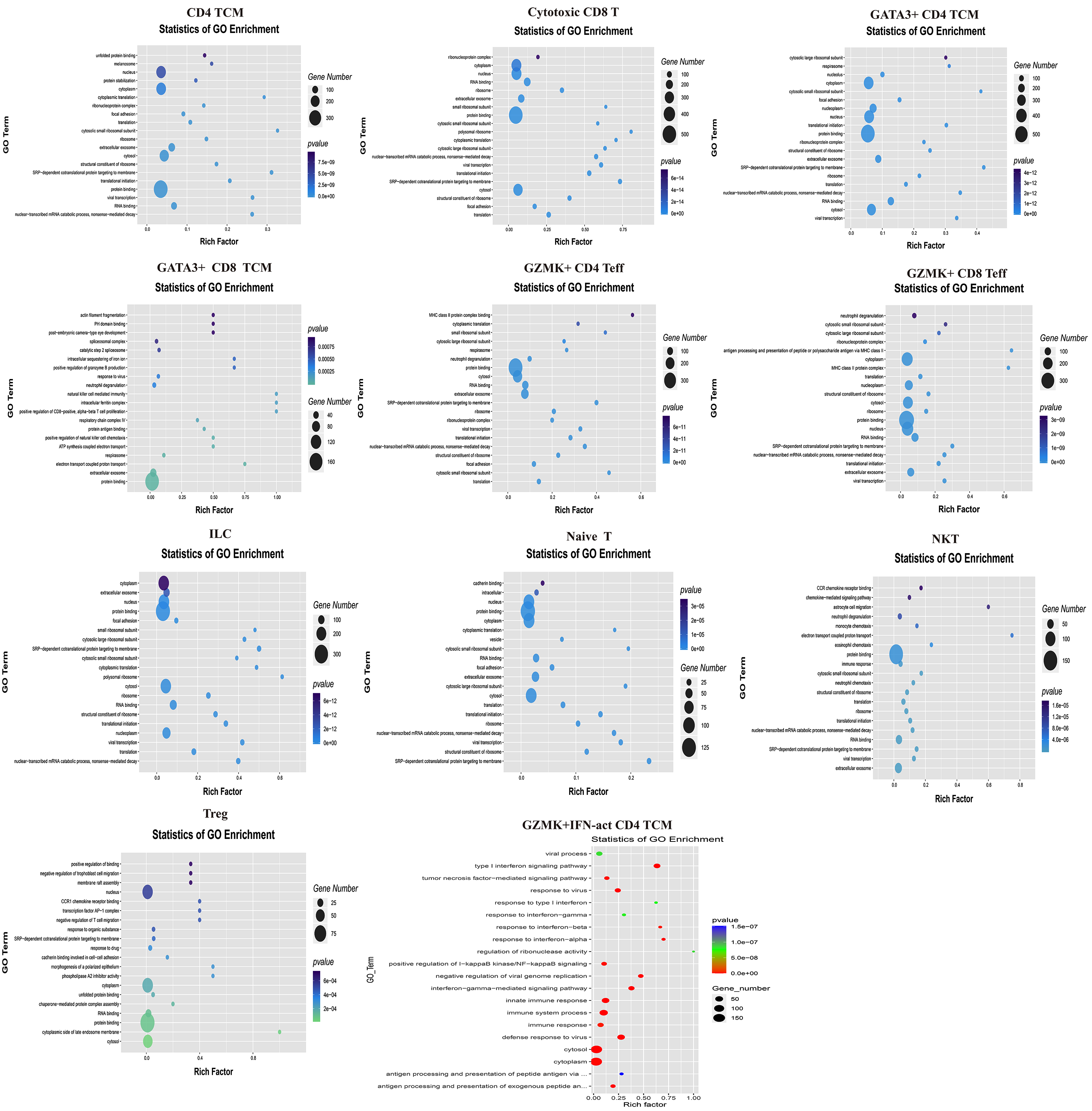

Supplement: Supplementary Figure 1 — Gene Ontology (GO) terms of T cell subtypes. (TCM=central memory T cell; Teff=effector T cell; Treg=regulator T cell; ILC=innate lymphoid cells; NKT=nature killer T cell) [file Image_1.tif]

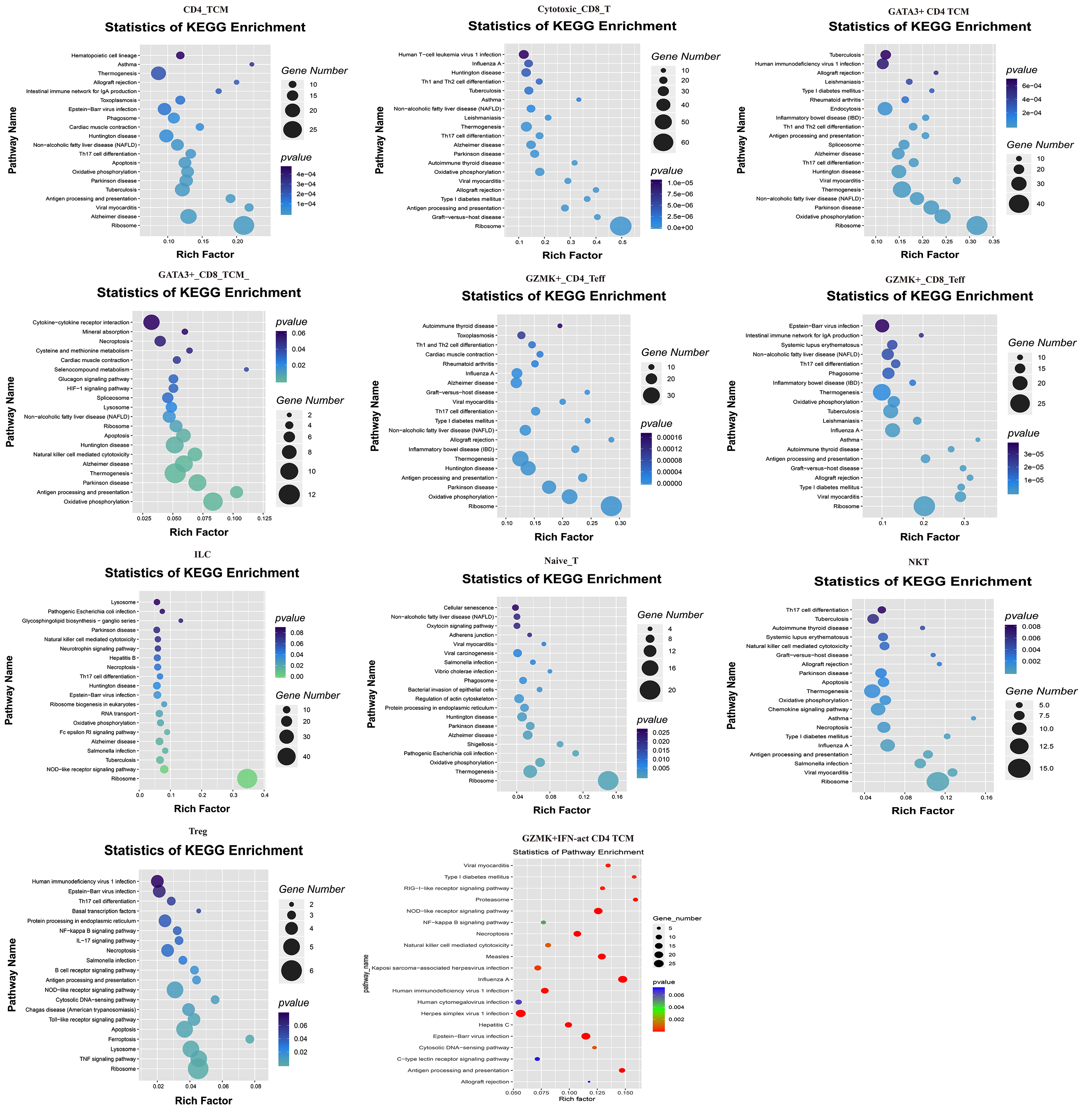

Supplement: Supplementary Figure 2 — Kyoto Encyclopedia of Genes and Genomes (KEGG) pathways of T cell subtypes. (TCM=central memory T cell; Teff=effector T cell; Treg=regulator T cell; ILC=innate lymphoid cells; NKT=nature killer T cell) [file Image_2.tif]

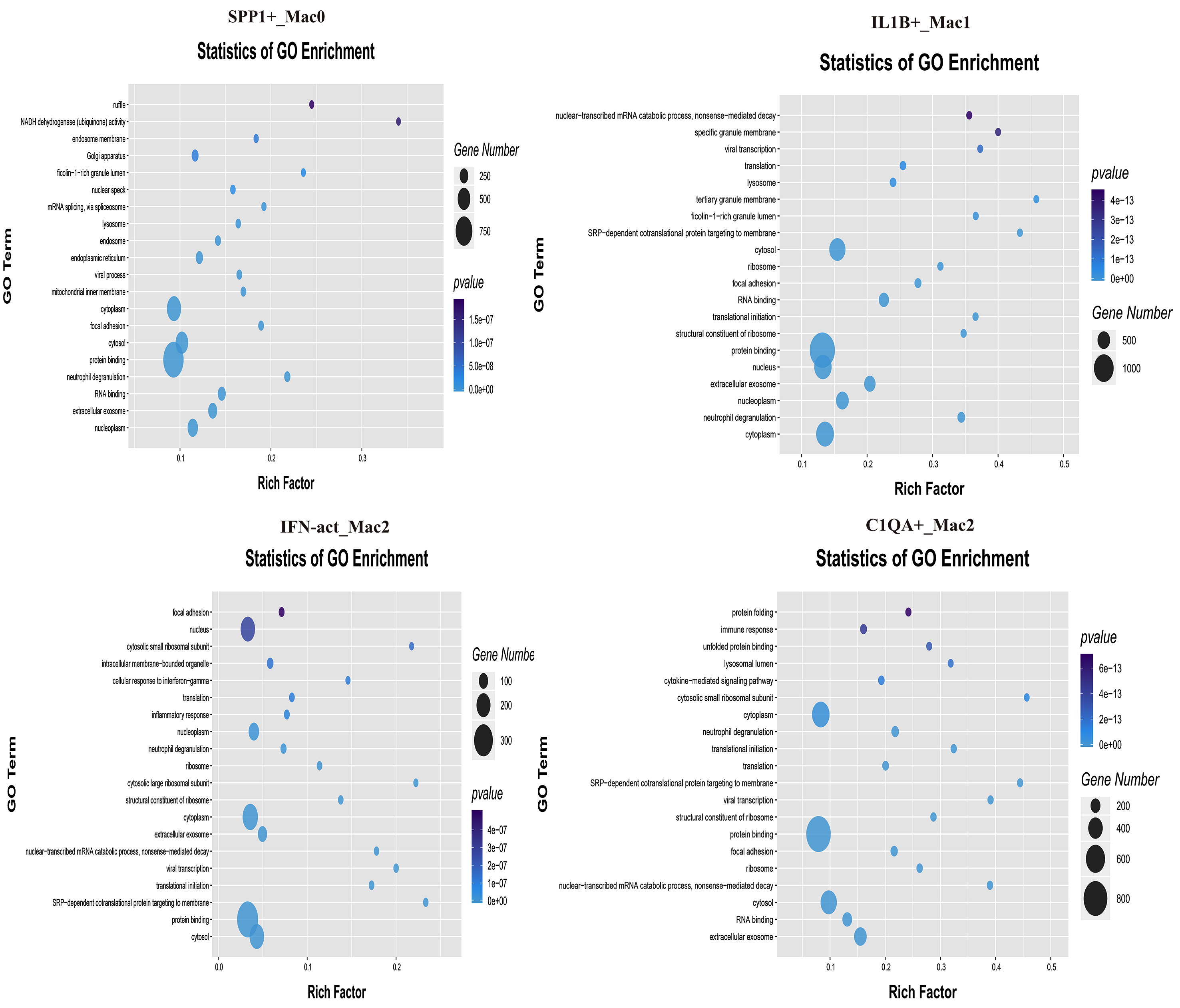

Supplement: Supplementary Figure 3 — Gene Ontology (GO) terms of macrophage (Mac) subtypes. [file Image_3.tif]

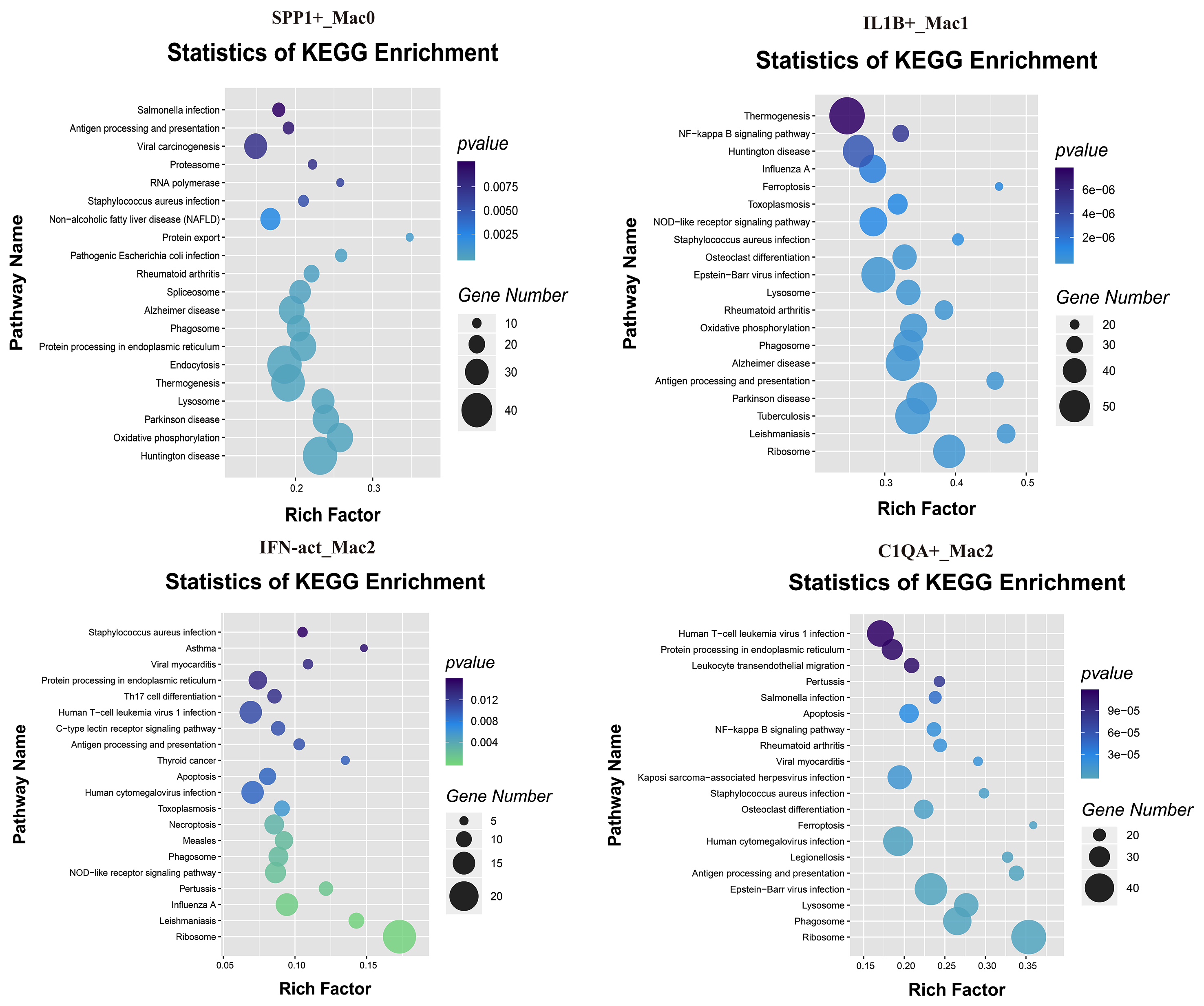

Supplement: Supplementary Figure 4 — Kyoto Encyclopedia of Genes and Genomes (KEGG) pathways of macrophage (Mac) subtypes. [file Image_4.tif]

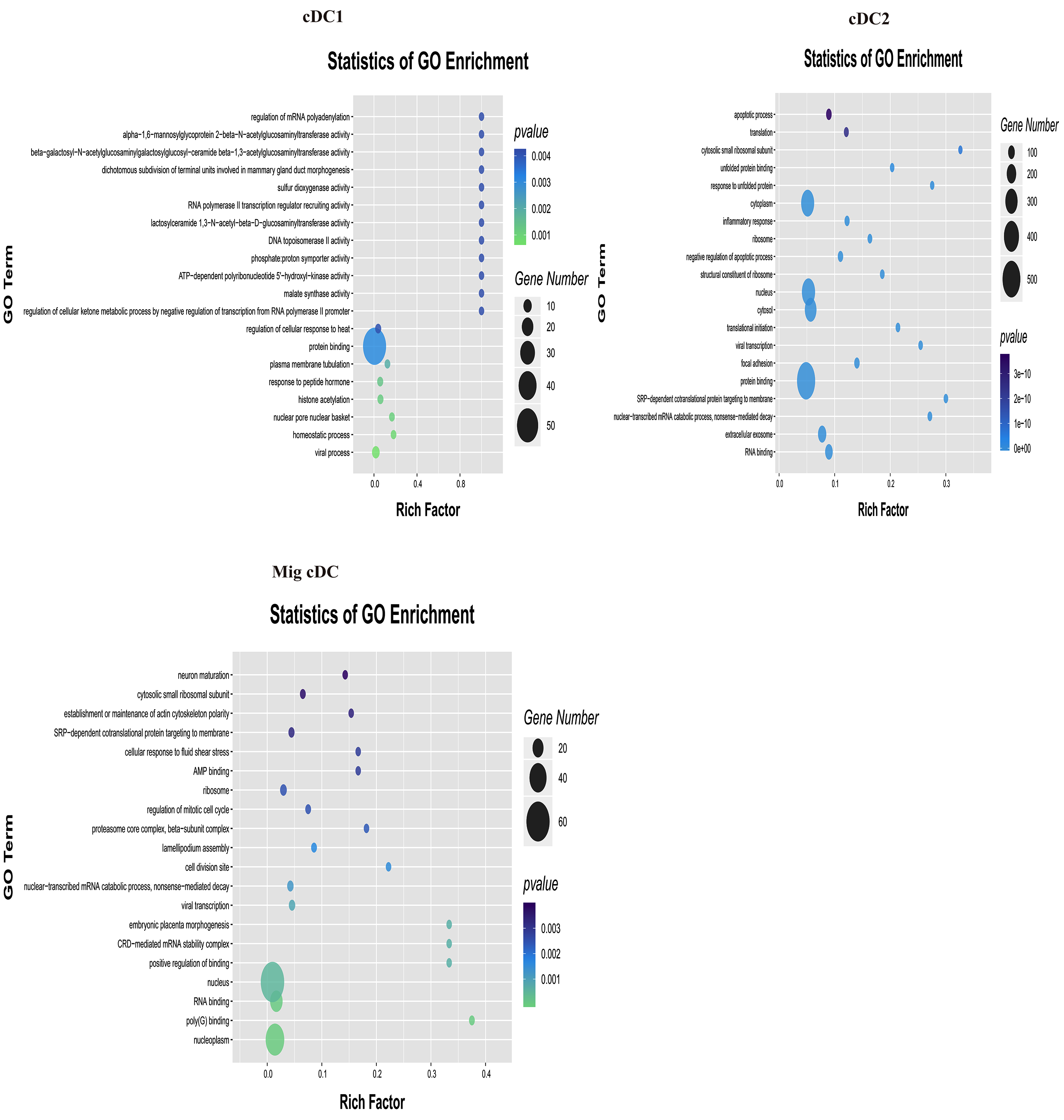

Supplement: Supplementary Figure 5 — Gene Ontology (GO) terms of dendritic cells (DCs) subtypes. [file Image_5.tif]

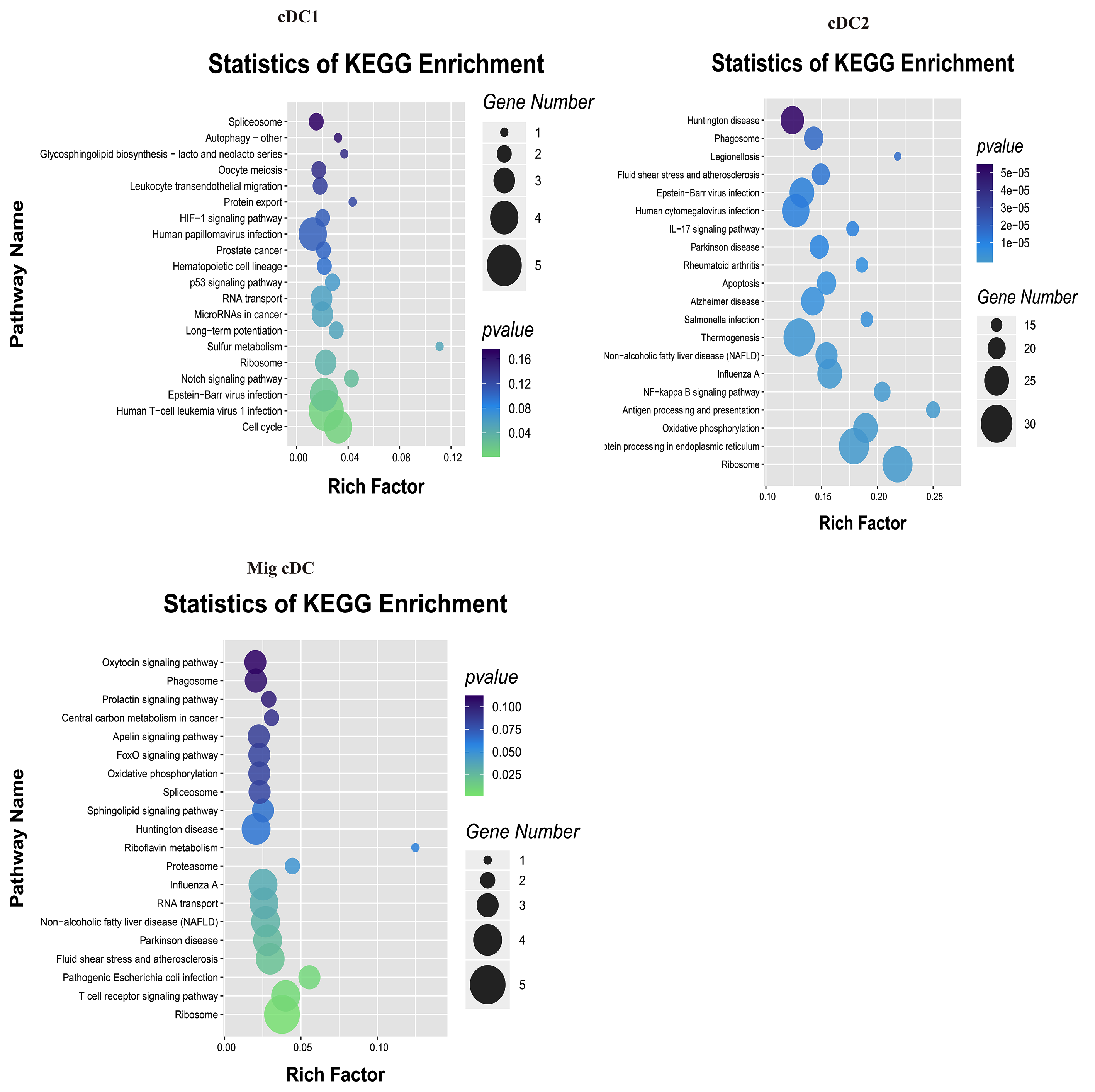

Supplement: Supplementary Figure 6 — Kyoto Encyclopedia of Genes and Genomes (KEGG) pathways of dendritic cells (DCs) subtypes. [file Image_6.tif]

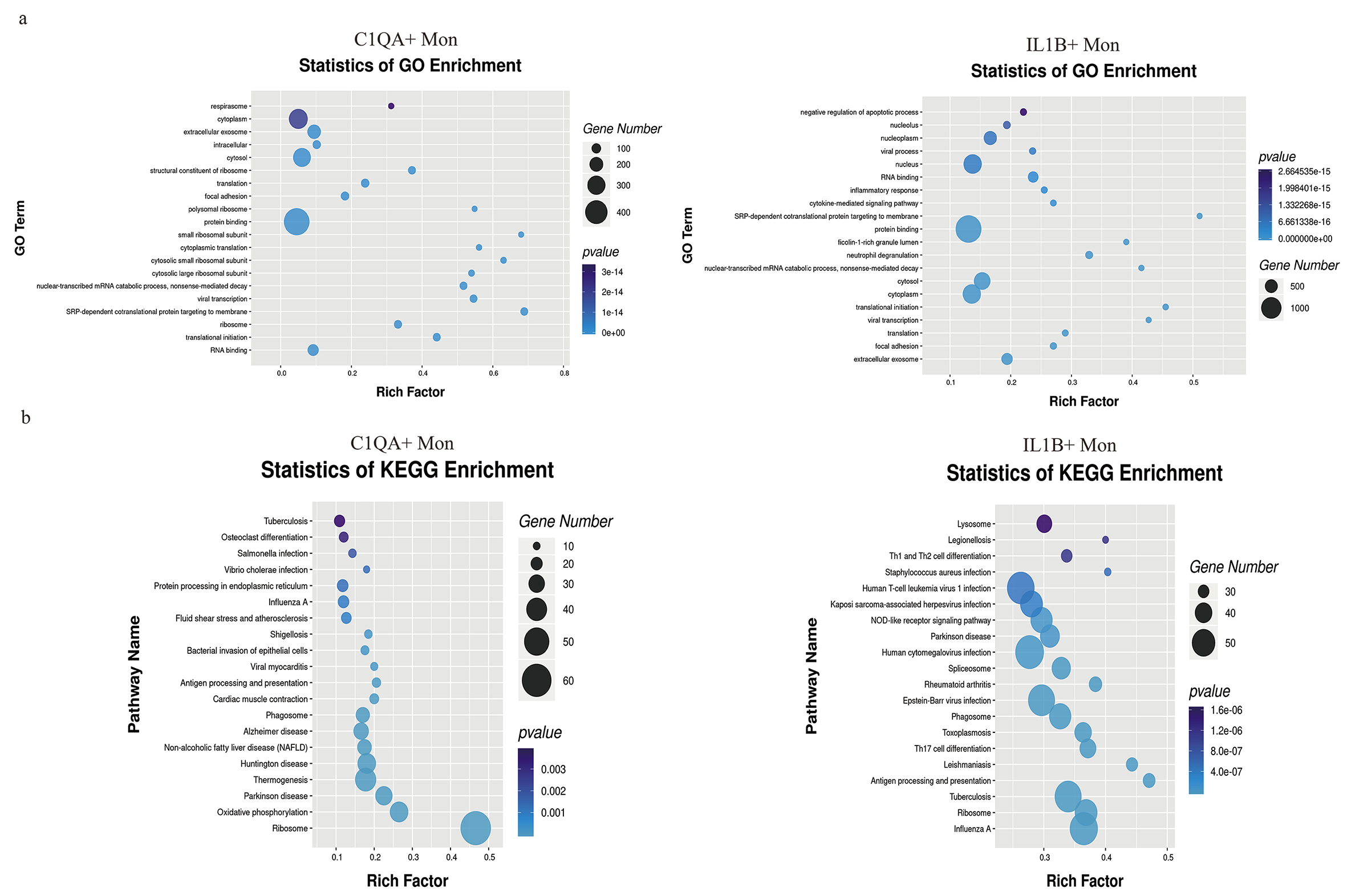

Supplement: Supplementary Figure 7 — Gene Ontology (GO) terms and Kyoto Encyclopedia of Genes and Genomes (KEGG) pathways of monocyte (Mon) subtypes. (A) GO terms of Mon subtypes. (B) KEGG pathways of Mon subtypes. [file Image_7.tif]

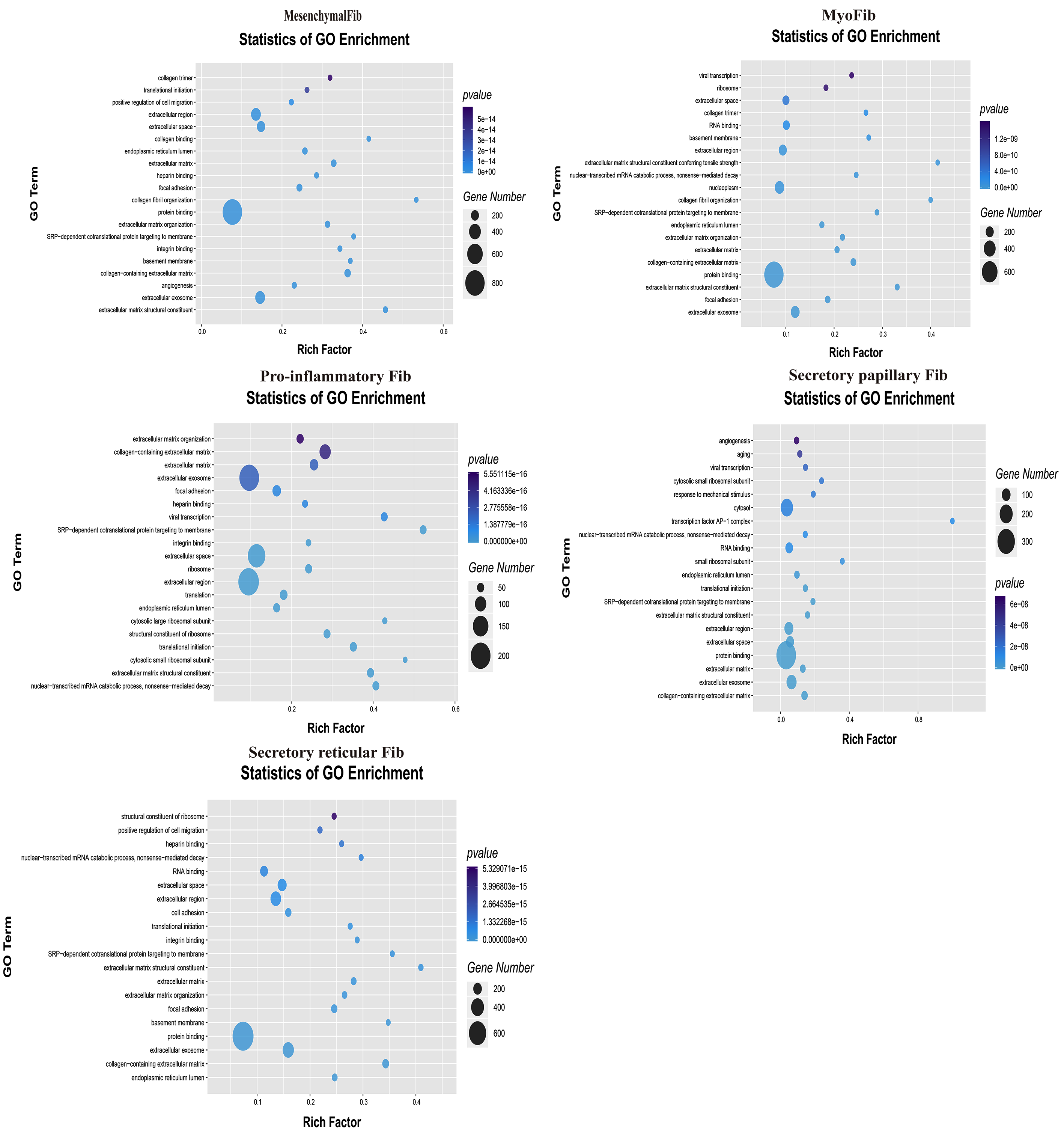

Supplement: Supplementary Figure 8 — Gene Ontology (GO) terms of fibroblast (Fib) subtypes. [file Image_8.tif]

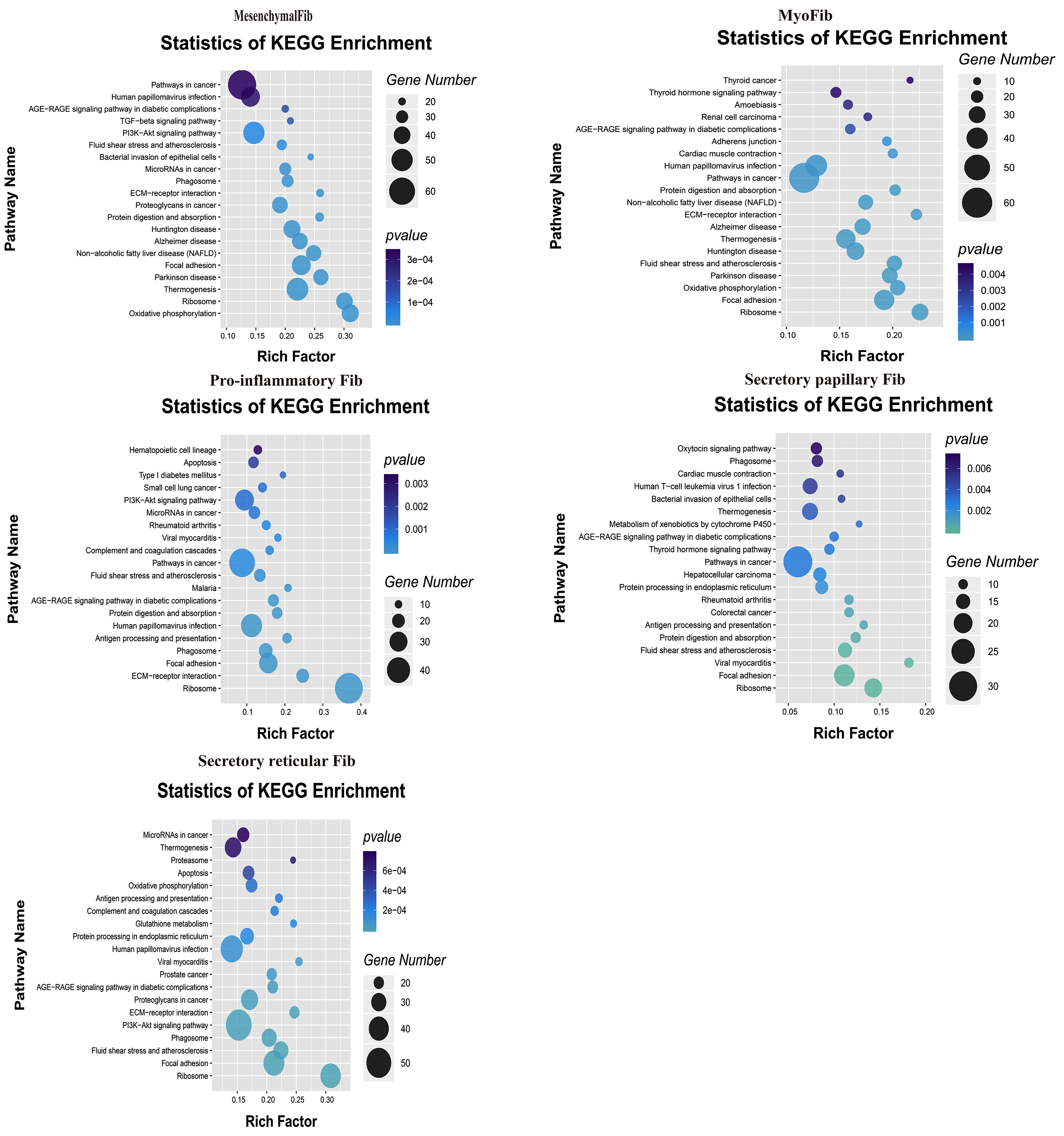

Supplement: Supplementary Figure 9 — Kyoto Encyclopedia of Genes and Genomes (KEGG) pathways of fibroblast (Fib) subtypes. [file Image_9.tif]
