# Supplementary material for: Single-cell RNA-seq reveals cellular heterogeneity from deep fascia in patients with acute compartment syndrome
Source: Front Immunol. 2023 Jan 18;13:1062479. doi: 10.3389/fimmu.2022.1062479 (PMC9889980; doi:10.3389/fimmu.2022.1062479)
Supplement: Supplementary file 10 [file Table_1.doc]

**Supplementary Table 1. Patients basic information**

| ***No of Patient*** | ***Age(years)*** | ***Sex*** | ***Diagnosis*** | ***Group*** | ***Sample source*** | ***Skin surface pressure*** |
| --- | --- | --- | --- | --- | --- | --- |
| 1 | 27 | female | tibiofibular fracture | HG | inside of middle shank | 68mmHg |
| 2 | 29 | male | tibiofibular fracture | HG | inside of middle shank | 72mmHg |
| 3 | 31 | female | tibiofibular fracture | HG | inside of middle shank | 67mmHg |
| 4 | 18 | male | [osteosarcoma](javascript:;) | NG | inside of middle shank | 28mmHg |
| 5 | 19 | male | [osteosarcoma](javascript:;) | NG | inside of middle shank | 30mmHg |
| 6 | 21 | female | [osteosarcoma](javascript:;) | NG | inside of middle shank | 29mmHg |

*NG= normal stress group; HG= high stress group
