# Supplementary material for: Single-cell RNA-seq reveals cellular heterogeneity from deep fascia in patients with acute compartment syndrome
Source: Front Immunol. 2023 Jan 18;13:1062479. doi: 10.3389/fimmu.2022.1062479 (PMC9889980; doi:10.3389/fimmu.2022.1062479)
Supplement: Supplementary file 11 [file Table_2.doc]

**Supplementary Table 2. Proportion and marker genes of all cell clusters or subclusters in two groups.**

| ***Cell Clusters***  ***Groups*** | ***NG*** | ***HG*** | ***p-value*** | ***Marker genes*** |
| --- | --- | --- | --- | --- |
| B cell（%） | 0.12 | 4.09 | ＜0.0001 | *CD79, CD19, MS4A1* |
| Cycling（%） | 0.26 | 1.76 | ＜0.0001 | *TOP2A, MKI67* |
| Endothelial（%） | 28.59 | 9.34 | ＜0.0001 | *VWF, PECAM1, CD34* |
| Fibroblast（%） | 28.98 | 8.49 | ＜0.0001 | *COL1A1, COL1A2, DCN* |
| Mast（%） | 1.41 | 1.97 | ＜0.0001 | *KIT, TPSB2* |
| Myeloid（%） | 6.70 | 14.53 | ＜0.0001 | *FCGR2A, CD33, ITGAM* |
| T cell（%） | 3.41 | 31.26 | ＜0.0001 | *CD3G, CD3E, CD3D* |
| SMC（%） | 30.47 | 26.82 | ＜0.0001 | *CNN1, ACTA2, TAGLN* |
| Other（%） | 0.06 | 1.75 | ＜0.0001 | -- |
| ***T cell subclusters*** | | | |  |
| CD4 TCM (%) | 8.35 | 16.13 | ＜0.001 | *CD4, TCF7, CD3D, CD3E, SELL, CCR7, GPR183, TCF7, ISG15* and *IL7R* |
| Cytotoxic CD8 T (%) | 13.09 | 2.98 | ＜0.001 | ISG15, *GNLY, GZMB, FCGR3A, NKG7, TRDC*, CD3E, CD8A and *CD3D* |
| GATA3^+^CD4 TCM (%) | 8.80 | 17.43 | ＜0.001 | *GATA3*, *TCF7, SELL, ISG15, CCR7, CD3D, CD3E, GZMK*, *GPR183,* and *IL7R* |
| GATA3^+^CD8 TCM (%) | 1.81 | 5.46 | ＜0.001 | *CD4, GATA3, SELL, CCR7, SELL, IL7R, GPR183, TCF7, GATA3, ISG15,* *CD8A, CD3D,* and *CD3E* |
| GZMK^+^CD4 Teff (%) | 33.97 | 32.47 | 0.363 | *CD4, TCF7, CD3D, CD3E, GZMK*, *GATA3, ISG15, GPR183,* and *IL7R* |
| GZMK^+^CD8 Teff (%) | 19.07 | 14.15 | ＜0.001 | *NKG7, IL7R, GPR183, TCF7, ISG15, GZMK*, *CD8A, CD3D,* and *CD3E* |
| GZMK^+^IFN-act CD4 TCM (%) | 0.11 | 1.01 | 0.008 | *ISG15, IFI6, CD4, GATA3*, *TCF7, CD3D, CD3E, GZMK*, *GPR183, CCR7, SELL* and *IL7R* |
| ILC (%) | 8.01 | 0.78 | ＜0.001 | *GATA3, GPR183, TCF7, GATA7, ISG15, IF16, IL7R, and TRDC* |
| Naive T (%) | 2.03 | 2.27 | 0.645 | *CD3D, CD3E, TCF7,* and *IL7R* |
| NKT (%) | 3.95 | 4.83 | 0.242 | GNLY, *GZMK, TRDC, TRGC1, GPR183, TCF7, GATA3, ISG15, IL7R, NKG7, SELL, CD3E* and *TRGC1* |
| Treg (%) | 0.79 | 2.48 | ＜0.001 | *FOXP3,* *CD4, CCR7, SELL, IL7R, GPR183, GATA3, ISG15, TCF7, CD3D,* and *CD3E* |
| ***Myeloid cell subclusters*** | | | |  |
| C1QA^+^Mon (%) | 2.07 | 8.01 | ＜0.001 | *VCAN, CD14, IL1B, C1QA, MSR1, MRC1, CD163* |
| IL1B^+^Mon (%) | 40.25 | 20.05 | ＜0.001 | *VCAN, CD14, IL1B, C1QA, MSR1, MRC1, CD163* |
| SPP1^+^Mac0 (%) | 0.69 | 14.72 | ＜0.001 | *VCAN, FCN1, CD14, NUPR1, SPP1, IL1B, IF16, C1QA, CD68, MSR1, MRC1, CD163* |
| IL1B^+^Mac1 (%) | 34.85 | 5.96 | ＜0.001 | *VCAN, FCN1, CD14, IL1B, IF16, CD68, MSR1, MRC1, CD163* |
| C1QA^+^Mac2 (%) | 15.99 | 27.77 | ＜0.001 | *VCAN, FCN1, CD14, IL1B, IF16, C1QA, CD68, MSR1, MRC1, CD163* |
| IFN-act Mac2 (%) | 0.69 | 5.70 | ＜0.001 | *VCAN, FCN1, CD14, IL1B, IF16, C1QA, CD68, MSR1, MRC1, CD163* |
| cDC1 (%) | 0.23 | 2.18 | ＜0.001 | *XCR1, CLEC9A* |
| cDC2 (%) | 3.85 | 14.32 | ＜0.001 | *VCAN, FCN1, CD14, IL1B, IF16, C1QA, MSR1, MRC1, CD163, FCER1A, CD1C* |
| Mig cDC (%) | 0.23 | 0.68 | 0.032 | *VCAN, IL1B, IF16, CD1C, CCL12, CCR7* |
| ***Fibroblast subclusters*** | | | |  |
| Mesenchymal Fib (%) | 14.34 | 22.71 | ＜0.001 | *MFAP5, ANGPTL1, CCN5, COL18A1, CXCL12, COMP, ASPN, COL11A1, COL1A1, DCN* |
| MyoFib (%) | 0.39 | 1.39 | ＜0.001 | *MFAP5, ANGPTL1, CCN5, COL18A1, CXCL12, RGS5, CCL19, APOE, RGS5, ACTA2, ASPN, COL1A1, DCN* |
| Pro-inflammatory Fib (%) | 45.21 | 48.76 | 0.003 | *MFAP5, ANGPTL1, CCN5, COL18A1, CXCL12, RGS5, APOE, ACTA2, ASPN, COL1A1, DCN* |
| Secretory papillary Fib (%) | 1.50 | 10.32 | ＜0.001 | *MFAP5, COL18A1, CXCL12, APOE, COL1A1, DCN* |
| Secretory reticular Fib (%) | 38.56 | 16.82 | ＜0.001 | *MFAP5, ANGPTL1, CCN5, COL18A1, CXCL12, ASPN, COL1A1, DCN* |

NG= normal stress group; HG= high stress group. P values were calculated by the chi-square test
